# Modeling the Variance of Passive SiPMs in the Nonlinear Regime

**DOI:** 10.3390/s26175579

**Published:** 2026-09-02

**Authors:** Víctor Moya, Jaime Rosado

**Affiliations:** IPARCOS and Department of EMFTEL, Universidad Complutense de Madrid, E-28040 Madrid, Spain

**Keywords:** silicon photomultipliers, SiPM, nonlinearity, resolution, Monte Carlo simulation, correlated noise

## Abstract

Silicon photomultipliers (SiPMs) are widely used in high-energy physics, medical imaging, and other photon-counting applications. While their nonlinear response at high light intensities is well known, its impact on the statistical fluctuations of the detector output remains much less understood. In this work, we develop an analytical framework for the variance of the charge response of passive-quenching SiPMs in the two limiting cases of instantaneous light pulses and pulses much longer than the pixel recovery time. Based on these exact results, we propose a phenomenological model that describes the variance of the SiPM charge response for arbitrary pulse durations while accounting for pixel recovery and correlated noise. The resulting framework is then used to predict the photon-counting resolution over the full dynamic range of the detector. The model is validated through Monte Carlo simulations and experimental measurements performed with laser, LED, and scintillation light sources. The results show that the optimal photon-counting resolution is generally reached well beyond the onset of nonlinear response, since pixel saturation introduces sub-Poissonian fluctuations that partially compensate for the nonlinear compression of the SiPM response. These findings provide a practical framework for predicting photon-counting resolution and optimizing the operation of passive SiPMs over a wide dynamic range.

## 1. Introduction

Silicon Photomultipliers (SiPMs) have become one of the leading technologies for low-light detection, offering a competitive alternative to traditional photomultiplier tubes (PMTs). They combine high gain, excellent timing resolution, and high photon detection efficiency (PDE) in the visible spectrum with compact size, relatively low operating voltages, immunity to magnetic fields, and low manufacturing costs [1]. These characteristics have led to their widespread adoption in high-energy physics [2,3,4,5,6], medical imaging [7,8,9], Compton cameras [10], LiDAR systems [11], and biophysics [12].

An SiPM consists of an array of avalanche photodiodes operating in Geiger mode, hereafter referred to as pixels [7]. Each pixel is reverse biased above its breakdown voltage so that an incident photon can trigger a self-sustaining avalanche. The probability that an incident photon triggers an avalanche is called photon detection efficiency, PDE, which is a function of both the wavelength and the applied overvoltage [13]. The charge released by a fully developed avalanche is essentially independent of the initial energy deposition and is determined mainly by the product of the pixel capacitance and the overvoltage. At a given overvoltage, the average avalanche charge defines the SiPM gain. Under ideal operating conditions, the mean output charge is expected to be proportional to the number of detected photons, while its variance is dominated by the fluctuations in the number of detected photons, together with the small excess noise associated with avalanche multiplication and device non-uniformities, the latter typically originating from pixel-to-pixel variations in electrical parameters.

In practice, several physical processes modify this ideal behavior. The most important are dark counts, correlated noise, and pixel recovery. Dark counts originate from thermally generated carriers that trigger avalanches in the absence of incident photons [14]. Since they are unrelated to previous avalanches, they constitute an uncorrelated noise source and are often negligible in measurements synchronized with short light pulses.

Correlated noise arises when a primary avalanche induces one or more secondary avalanches through internally generated photons or charge carriers [13,15,16]. If a neighboring pixel is triggered, the process is referred to as crosstalk, which may occur promptly or after a short delay. Alternatively, if the original pixel is re-triggered after partial recovery, the effect is known as afterpulsing. Owing to their stochastic nature, these processes introduce additional fluctuations, which are commonly described through an excess noise factor [17,18].

Another key effect governing the SiPM response is pixel recovery. In passive-quenching SiPMs, after an avalanche, a pixel requires a finite recharge time during which its overvoltage recovers, typically following an exponential law characterized by a recovery time τ [7]. Consequently, photons arriving before full recovery generate avalanches with reduced probability and charge, producing a nonlinear relationship between the measured signal and the number of incident photons. Since photon arrival times are themselves stochastic, the recovery process also introduces an additional contribution to the signal variance.

The importance of this nonlinearity depends strongly on the ratio between the pulse duration and the recovery time. For light pulses much shorter than τ, nonlinear effects become significant as the number of photoelectrons approaches the total number of pixels *N*. For instance, when the number of photoelectrons reaches approximately 20% of *N*, the measured charge is already about 10% lower than that expected from an ideal linear response, showing that nonlinear effects become relevant well before saturation is reached [19]. In contrast, for pulses much longer than τ, pixels can recharge between successive interactions and the response remains approximately linear up to much higher photon fluxes, with noticeable nonlinear effects appearing only when the number of photoelectrons exceeds *N*. Pixel recovery also suppresses correlated-noise processes such as crosstalk by reducing the number of fully charged neighboring pixels available to sustain secondary avalanches [19].

While the average nonlinear response of SiPMs has been extensively investigated [19,20,21,22,23,24], considerably less attention has been devoted to understanding how nonlinearity modifies the statistical variance of the detector response. This question has become increasingly relevant as substantial effort is being devoted to extending the dynamic range of SiPMs through nonlinear-response corrections [25,26,27]. Since photon-counting resolution is ultimately determined by the signal variance, accurate variance models are required to assess the achievable performance.

Some existing analytical treatments of the signal variance are restricted to limiting cases such as instantaneous light pulses [28] or to the weakly nonlinear regime [29], because the effect of pixel recovery between successive impinging photons, combined with correlated noise, makes a general analytical solution intractable. S. Vinogradov et al. estimated the stochastic losses of detected photons during pixel recovery on the basis of a non-paralyzable dead-time model [21]. This may be adequate for active-quenching digital SiPMs [30], where dedicated electronics rapidly quench each avalanche and restore the pixel to its operating voltage with a well-defined non-paralyzable dead time, but not for conventional passive-quenching SiPMs. The model presented in [21] was improved in [31] to partially account for the recovery process in passive SiPMs. However, a more general model valid for a wide range of photon fluxes and pulse durations is still needed.

In this work, we develop a model for the statistical variance of passive SiPMs that remains applicable over the full operating range, from the linear regime to strong saturation. We first derive exact analytical expressions for two limiting cases: instantaneous light pulses, for which pixel recovery is negligible, and long pulses, for which pixels fully recover between successive interactions. We then extend the model to arbitrary pulse durations through a phenomenological interpolation that incorporates correlated-noise effects. The model is validated using both Monte Carlo simulations, based on a modified version of the framework presented in [32], and compared with the previous models presented by S. Vinogradov et al. Finally, our model is validated with experimental measurements performed with laser, pulsed-LED, and scintillation signals.

## 2. Theoretical Framework

Under suitable simplifying assumptions, the SiPM response can be described analytically. We first derive exact expressions for two limiting cases: instantaneous light pulses and very long light pulses. We then extend the treatment to real operating conditions by introducing a phenomenological model that accounts for pixel recovery and correlated noise. Finally, we derive the corresponding photon-counting resolution.

### 2.1. Instantaneous Light Pulses

We first consider the limiting case of instantaneous light pulses, for which pixel recovery can be neglected. Since each pixel can fire at most once during the pulse, the SiPM response admits an exact analytical treatment.

The total charge released by the SiPM in response to a light pulse is

(1) $$Q=\sum_{i=1}^{n_{f}}q_{i},$$

where $n_{f}$ is the number of fired pixels and $q_{i}$ is the charge released by the *i*-th pixel. Because correlated noise is neglected and the light pulse is assumed to be instantaneous, each pixel can fire at most once. Consequently, $n_{f}$ is equal to the total number of avalanches. The random variables $q_{i}$ are assumed to be independent and identically distributed, with mean *q* and variance $s^{2}$. Under these assumptions,

(2) $$\begin{array}{cc} E[Q] & =E\left[E\left[Q|n_{f}\right]\right]=q\cdot E\left[n_{f}\right]\;, \end{array}$$

(3) $$\begin{array}{cc} \mathrm{Var}[Q] & =E\left[\mathrm{Var}\left[Q|n_{f}\right]\right]+\mathrm{Var}\left[E\left[Q|n_{f}\right]\right]=s^{2}\cdot E\left[n_{f}\right]+q^{2}\cdot \mathrm{Var}\left[n_{f}\right]. \end{array}$$

The parameters *q* and *s* can be obtained experimentally from the single-photon charge spectrum (see Section 5.1). To relate the number of fired pixels to the incident light, it is convenient to introduce the intermediate random variable $n_{s}$, referred to as the number of *avalanche seeds* [19]. For a given number of incident photons $n_{\mathrm{ph}}$, $n_{s}$ follows the binomial distribution

(4) P(ns|nph)=nphns·PDEns·(1−PDE)nph−ns.

In the linear regime ($n_{s}\ll N$), each avalanche seed occupies a different pixel, so that $n_{f}=n_{s}$. As the light intensity increases, however, several seeds may fall within the same pixel. Since an instantaneous pulse allows each pixel to fire only once, the number of fired pixels satisfies $n_{f}\leq n_{s}$.

For a fixed number of seeds, the problem reduces to counting the possible ways in which the seeds can be distributed among the *N* pixels. There are $N^{n_{s}}$ possible distributions in total. If exactly $n_{f}$ pixels are occupied, the $n_{s}$ seeds can be partitioned into these pixels in $S(n_{s},n_{f})$ ways, where $S(n_{s},n_{f})$ denotes a Stirling number of the second kind. These partitions can be assigned to the occupied pixels in $n_{f}!$ different ways, while the occupied pixels themselves can be selected from the *N* available pixels in Nnf ways. Therefore,

(5) P(nf|ns)=Nnf·nf!·S(ns,nf)Nns=N!·S(ns,nf)(N−nf)!·Nns,

whose first two moments are

(6) $$\begin{array}{cc} E[n_{f}|n_{s}] & =N\cdot \left[1-{\left(1-\frac{1}{N}\right)}^{n_{s}}\right], \end{array}$$

(7) $$\begin{array}{cc} \mathrm{Var}[n_{f}|n_{s}] & =N\cdot {\left(1-\frac{1}{N}\right)}^{n_{s}}+N\cdot \left(N-1\right)\cdot {\left(1-\frac{2}{N}\right)}^{n_{s}}-{\left[N\cdot {\left(1-\frac{1}{N}\right)}^{n_{s}}\right]}^{2}. \end{array}$$

The unconditional distribution of the number of fired pixels is obtained by marginalizing over the number of avalanche seeds,

(8) $$P\left(n_{f}\right)=\sum_{n_{s}=0}^{\infty }P\left(n_{f}|n_{s}\right)\cdot P\left(n_{s}\right),$$

where

(9) $$P\left(n_{s}\right)=\sum_{n_{\mathrm{ph}}=0}^{\infty }P\left(n_{s}|n_{\mathrm{ph}}\right)\cdot P\left(n_{\mathrm{ph}}\right).$$

For Poisson-distributed incident photons, the thinning property of the Poisson distribution implies that the number of avalanche seeds is also Poisson distributed, with $E\left[n_{s}\right]=\mathrm{Var}\left[n_{s}\right]=PDE\cdot E\left[n_{\mathrm{ph}}\right]$. Consequently,

(10) $$\begin{array}{cc} E[n_{f}] & =N\cdot \left(1-e^{-x}\right), \end{array}$$

(11) $$\begin{array}{cc} \mathrm{Var}[n_{f}] & =N\cdot \left(1-e^{-x}\right)\cdot e^{-x}, \end{array}$$

where we define the dimensionless parameter

(12) $$x=\frac{PDE\cdot E[n_{\mathrm{ph}}]}{N}=\frac{E[n_{s}]}{N},$$

which represents the average number of avalanche seeds per pixel.

Notably, Equations (10) and (11) coincide with the first two moments of the binomial distribution

(13) P(nf)=Nnf·pnf·(1−p)N−nf,

with $p=1-e^{-x}$, which is the approximation adopted in [28]. However, the exact distribution given by Equations (5) and (8) is not identical to the binomial distribution, since they differ in their higher-order moments.

Substituting Equations (10) and (11) into Equations (2) and (3) yields

(14) $$\begin{array}{cc} E[Q] & =N\cdot q\cdot \left(1-e^{-x}\right), \end{array}$$

(15) $$\begin{array}{cc} \mathrm{Var}[Q] & =N\cdot q^{2}\cdot \left(1-e^{-x}\right)\cdot \left(e^{-x}+\frac{s^{2}}{q^{2}}\right). \end{array}$$

To obtain a characterization of the detector response independent of the total number of pixels *N*, we consider the normalized mean charge $\frac{E[Q]}{N\cdot q}$ and scale the charge resolution $\frac{\sqrt{\mathrm{Var}[Q]}}{E[Q]}$ by a factor of $\sqrt{N}$, yielding

(16) $$\begin{array}{cc} \frac{E[Q]}{N\cdot q} & =1-e^{-x}, \end{array}$$

(17) $$\begin{array}{cc} \frac{\sqrt{N\cdot \mathrm{Var}[Q]}}{E[Q]} & =\sqrt{\frac{e^{-x}+\frac{s^{2}}{q^{2}}}{1-e^{-x}}}=\sqrt{\frac{1+\frac{s^{2}}{q^{2}}}{1-e^{-x}}-1}, \end{array}$$

where $1+s^{2}/q^{2}$ corresponds to the excess noise factor associated with avalanche multiplication and the electronic readout.

In the linear regime (x≪1), where $n_{f}=n_{s}$, these expressions reduce to

(18) $$\begin{array}{cc} \frac{E[Q]}{N\cdot q} & =x, \end{array}$$

(19) $$\begin{array}{cc} \frac{\sqrt{N\cdot \mathrm{Var}[Q]}}{E[Q]} & =\sqrt{\frac{1+\frac{s^{2}}{q^{2}}}{x}}, \end{array}$$

which are independent of the pulse duration.

In the opposite limit (x→∞), all pixels fire and

(20) $$\begin{array}{cc} \frac{E[Q]}{N\cdot q} & =1, \end{array}$$

(21) $$\begin{array}{cc} \frac{\sqrt{N\cdot \mathrm{Var}[Q]}}{E[Q]} & =\frac{s}{q}, \end{array}$$

so that the charge resolution corresponds to the intrinsic fluctuations in *N* independent avalanche charges.

**Figure 1:**
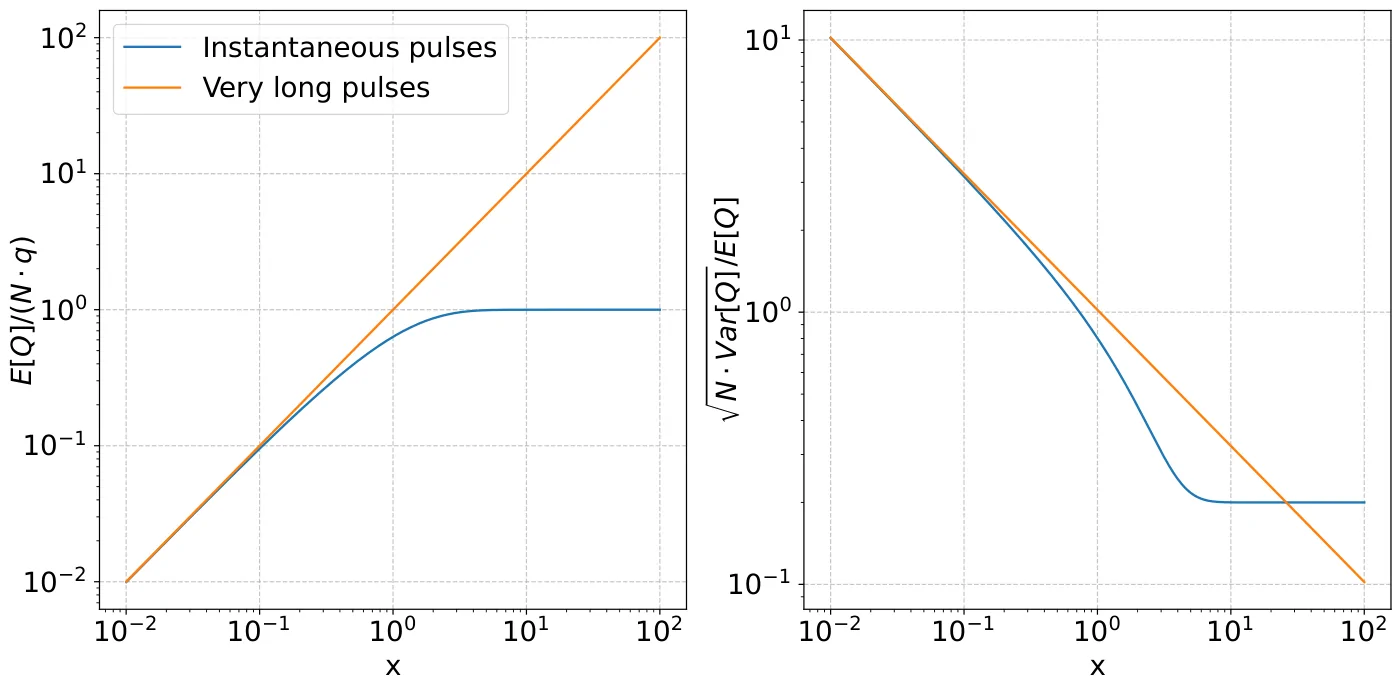
Exact solutions for $\frac{E[Q]}{N\cdot q}$ (**left-hand panel**) and $\frac{\sqrt{N\cdot \mathrm{Var}[Q]}}{E[Q]}$ (**right-hand panel**) as functions of $x=PDE\cdot E[n_{\mathrm{ph}}]/N$ for the limiting cases of instantaneous light pulses and very long light pulses (complete pixel recovery). A value of s/q=0.2 for the intrinsic avalanche-charge fluctuations is assumed.

Figure 1 shows expressions (16) and (17) for s/q=0.2. As saturation develops, $\frac{\sqrt{N\cdot \mathrm{Var}[Q]}}{E[Q]}$ becomes smaller than that expected for an ideal linear detector because the distribution of fired pixels becomes sub-Poissonian. Only in the asymptotic saturation limit does this quantity approach the intrinsic avalanche-charge fluctuations, s/q. This reduction in statistical fluctuations partially compensates for the nonlinear compression of the SiPM response, an effect that will prove important when analyzing the photon-counting resolution.

### 2.2. Very Long Light Pulses

The second limiting case corresponds to very long light pulses, for which the incident photons are distributed over a sufficiently long time interval *T* such that T≫x·τ. Under this condition, the average time between consecutive avalanche seeds impinging on the same pixel is much longer than the pixel recovery time, so each pixel fully recovers before another photon interacts with it. Consequently, every avalanche seed produces an avalanche with the full charge *q*, irrespective of previous events. Unlike the case of instantaneous light pulses, the total output charge is therefore no longer limited by the finite number of pixels *N*, but is directly proportional to the number of avalanche seeds,

(22) $$Q=\sum_{i=1}^{n_{s}}q_{i}\;.$$

Assuming that the avalanche charges are independent and identically distributed, with mean *q* and variance $s^{2}$, the first two moments of *Q* are

(23) $$\begin{array}{cc} E[Q] & =E\left[E\left[Q|n_{s}\right]\right]=q\cdot E\left[n_{s}\right], \end{array}$$

(24) $$\begin{array}{cc} \mathrm{Var}[Q] & =E\left[\mathrm{Var}\left[Q|n_{s}\right]\right]+\mathrm{Var}\left[E\left[Q|n_{s}\right]\right]=s^{2}\cdot E\left[n_{s}\right]+q^{2}\cdot \mathrm{Var}\left[n_{s}\right]. \end{array}$$

Assuming, as before, that the number of avalanche seeds follows a Poisson distribution with $E\left[n_{s}\right]=\mathrm{Var}\left[n_{s}\right]=N\cdot x$, the normalized mean charge and the scaled charge resolution become

(25) $$\begin{array}{cc} \frac{E[Q]}{N\cdot q} & =x, \end{array}$$

(26) $$\begin{array}{cc} \frac{\sqrt{N\cdot \mathrm{Var}[Q]}}{E[Q]} & =\sqrt{\frac{1+\frac{s^{2}}{q^{2}}}{x}}. \end{array}$$

These expressions are identical to (18) and (19), which describe the linear regime of instantaneous light pulses. Here, however, they remain valid over the entire range of *x*, provided that the condition T≫x·τ is satisfied.

The corresponding curves are shown in Figure 1, together with the exact solution for instantaneous light pulses. The comparison illustrates the role of pixel recovery: whereas instantaneous illumination leads to saturation as the number of avalanche seeds approaches the number of pixels, complete pixel recovery prevents saturation and preserves a linear response over a much wider dynamic range.

### 2.3. Response of an SiPM Under Real Conditions

Real SiPMs exhibit both correlated and uncorrelated noise, while their pixel recovery time is generally not negligible compared with the duration of the incident light pulse. Under these conditions, an exact analytical description of the SiPM response is no longer possible. Nevertheless, the theoretical framework developed in the previous subsections can be extended under a set of physically motivated approximations.

- Correlated noise generates secondary stochastic avalanches, thereby modifying the relationship between the number of impinging photons and the total output charge. Nevertheless, all expressions derived in Section 2.1 and Section 2.2 remain valid if $n_{f}$ and $n_{s}$ are interpreted as the number of fired pixels and avalanche seeds produced exclusively by impinging photons (i.e., excluding avalanches generated by correlated noise), provided that the avalanche charges $q_{i}$ in Equations (1) and (22), as well as their mean *q* and variance $s^{2}$ in Equations (2), (3), (23) and (24), are replaced by effective quantities that incorporate the contribution of correlated noise. In contrast, uncorrelated noise can generally be neglected when analyzing signals synchronized with short light pulses.
- For light pulses with non-negligible duration, successive avalanche seeds may arrive at pixels that have not yet fully recovered from previous avalanches. Both the probability of triggering an avalanche and the corresponding avalanche charge then depend on the recovery state of the pixel. As a consequence, the total output charge is neither limited by the number of pixels, as in Equation (1), nor strictly proportional to the number of avalanche seeds, as in Equation (22). The detector response is therefore expected to lie between the two limiting cases described by Equations (16), (17), (25) and (26), with the exact behavior depending on the temporal profile of the incident light pulse.

In Ref. [19], we proposed phenomenological models for the mean output charge that satisfy these requirements and accurately reproduce both simulated and experimental SiPM responses. Two pulse families were considered: exponential-like pulses and pulses with finite duration (e.g., rectangular pulses). For exponential-like pulses, the normalized mean charge is described by

(27) $$\frac{E[Q]}{N\cdot q}=\left[1+c\cdot e^{-d\cdot x}+a\cdot \ln\left(1+b\cdot x\right)\right]\cdot \left(1-e^{-x}\right),$$

whereas for pulses of finite duration,

(28) $$\frac{E[Q]}{N\cdot q}=\left[1+c\cdot e^{-d\cdot x}+\frac{a\cdot x}{1+b\cdot x}\right]\cdot \left(1-e^{-x}\right).$$

In both equations, the factor $1-e^{-x}$ represents the mean fraction of pixels triggered directly by primary impinging photons (10), while the factor in square brackets denotes the normalized mean charge released per triggered pixel. The latter consists of three distinct contributions: the primary avalanche (which contributes unity), a term accounting for subsequent avalanche triggers in the recovering pixel, a·ln(1+b·x) or $\frac{a\cdot x}{1+b\cdot x}$, and the term $c\cdot e^{-d\cdot x}$ accounting for correlated noise. This last term incorporates both correlated noise cascades and the reduced charge delivered by afterpulses. It decreases exponentially with increasing light intensity because the pool of fully recovered, available pixels is progressively depleted. The parameters *a*, *b*, *c*, and *d* are positive fitting parameters that depend on both the SiPM characteristics and the temporal profile of the incident light pulse.

These models can be interpreted as a generalization of expression (16) derived for instantaneous light pulses in the absence of correlated noise. The mean avalanche charge *q* of a fully recovered pixel is replaced by an effective mean charge per triggered pixel, corresponding to *q* times the factor in square brackets in Equations (27) and (28). For a=c=0, expression (16) is recovered.

In the linear regime (x≪1), both models reduce to

(29) $$\frac{E[Q]}{N\cdot q}=\left(1+c\right)\cdot x,$$

showing that the factor q·(1+c) corresponds to the effective gain of the SiPM. In the limit x→∞, expression (27) grows logarithmically, while expression (28) saturates to 1+a/b. The mean output charge for exponential-like pulses does not saturate because photons keep hitting the detector at arbitrarily late times.

**Figure 2:**
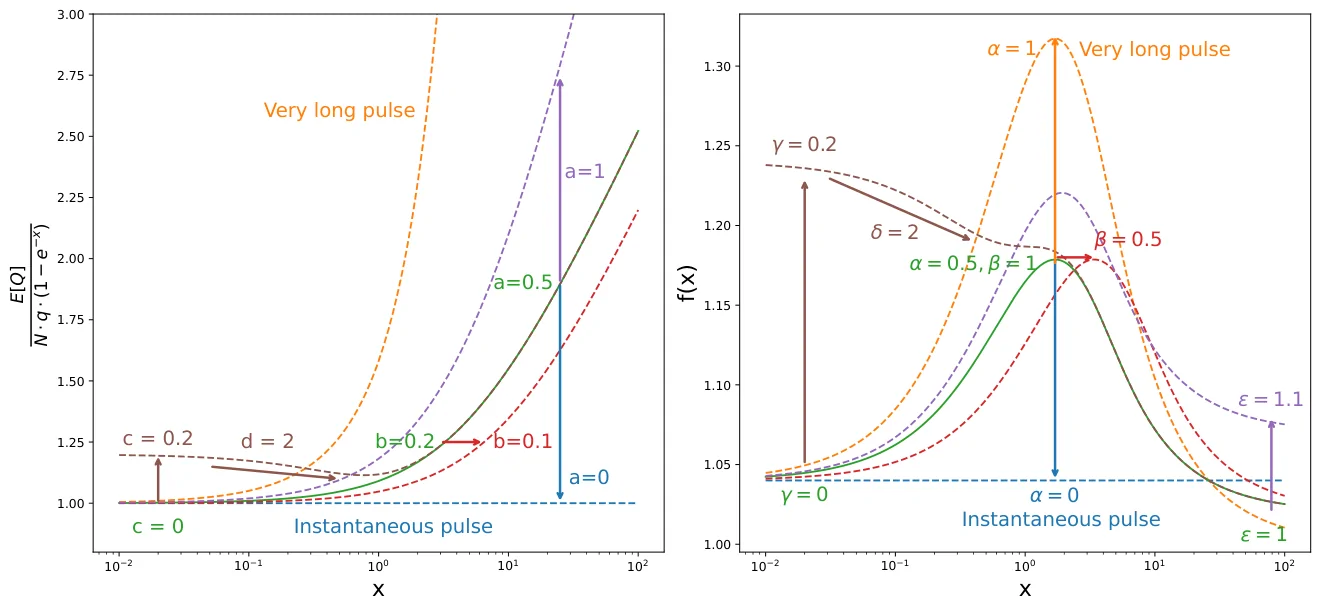
Normalized charge per fired pixel $\frac{E[Q]}{N\cdot q\cdot (1-e^{-x})}$ for exponential-like pulses (**left-hand panel**) and the function f(x) (**right-hand panel**) for different combinations of model parameters. The arrows indicate the effect of each parameter. The limiting cases of instantaneous and very long light pulses are also shown for comparison. A value of s/q=0.2 for the intrinsic avalanche-charge fluctuations is assumed.

The left-hand panel of Figure 2 shows several examples of the normalized charge per fired pixel for exponential-like pulses, i.e., the term in square brackets of expression (27). The mechanisms influencing the nonlinear response are better visualized in this representation, where the primary saturation trend $1-e^{-x}$ is factored out. The effect of the model parameters on the resulting curves is illustrated by arrows. In particular, *a* sets the overall amplitude of the contribution from photons arriving during pixel recovery, while *b* controls its rate of growth with light intensity. The parameter *d* controls the suppression rate of correlated noise with increasing *x*. The limiting cases of instantaneous and very long light pulses are also shown in the figure for comparison.

A similar strategy can be adopted to model the statistical variance of the SiPM response. Rather than modeling Var[Q] directly, we search for an expression that describes the scaled resolution of charge $\frac{\sqrt{N\cdot \mathrm{Var}[Q]}}{E[Q]}$, while keeping the previously derived model for $\frac{E[Q]}{N\cdot q}$ unchanged. This approach allows the mean response and its statistical fluctuations to be modeled independently. To do that, expression (17) is generalized by replacing the excess noise factor $1+\frac{s^{2}}{q^{2}}$ by a function f(x) accounting for the relative charge fluctuations per triggered pixel, including the effects of correlated noise and photons arriving during pixel recovery,

(30) $$\frac{\sqrt{N\cdot \mathrm{Var}[Q]}}{E[Q]}=\sqrt{\frac{f(x)}{1-e^{-x}}-1}.$$

The function f(x) is required to reproduce the two exact limiting cases. For instantaneous light pulses,

(31) $$f\left(x\right)=1+\frac{s^{2}}{q^{2}},$$

whereas for very long light pulses (T≫x·τ),

(32) $$f\left(x\right)=\left(1-e^{-x}\right)\cdot \left(1+\frac{1+\frac{s^{2}}{q^{2}}}{x}\right),$$

which follows directly by equating expressions (19) and (30).

Motivated by these restrictions on f(x), we propose the following expression for Poissonian light pulses:

(33) $$f\left(x\right)=\alpha \cdot \left(1-e^{-\beta \cdot x}\right)\cdot \left(\varepsilon +\frac{1+\frac{s^{2}}{q^{2}}+\gamma \cdot e^{-\delta \cdot x}}{\beta \cdot x}\right)+\left(1-\alpha \right)\cdot \left(1+\frac{s^{2}}{q^{2}}+\gamma \cdot e^{-\delta \cdot x}\right),$$

which can be interpreted as a phenomenological interpolation between the two exact limiting behaviors. The parameter α determines the relative weight of each limit, ranging from α=0 for instantaneous light pulses to α=1 for very long pulses. The term $\gamma \cdot e^{-\delta \cdot x}$ accounts for the relative charge fluctuations due to correlated noise in a similar way as the term $c\cdot e^{-d\cdot x}$ accounts for its mean charge contribution in Equations (27) and (28). The parameters β and ε are introduced to provide greater flexibility to f(x). While remaining close to unity, fine tuning β and ε allows the model to properly describe simulated and experimental data, as explained below.

In the linear regime (x≪1), expressions (33) and (30) reduce to

(34) $$\begin{array}{cc} f(x) & =1+\frac{s^{2}}{q^{2}}+\gamma , \end{array}$$

(35) $$\begin{array}{cc} \frac{\sqrt{N\cdot \mathrm{Var}[Q]}}{E[Q]} & =\sqrt{\frac{1+\frac{s^{2}}{q^{2}}+\gamma }{x}}. \end{array}$$

Expression (35) is identical to (19), except that the excess noise factor now includes the contribution from correlated noise through the parameter γ.

In the saturation limit (x→∞), assuming δ>0, the proposed model yields

(36) $$\begin{array}{cc} f(x) & =\alpha \cdot \varepsilon +\left(1-\alpha \right)\cdot \left(1+\frac{s^{2}}{q^{2}}\right), \end{array}$$

(37) $$\begin{array}{cc} \frac{\sqrt{N\cdot \mathrm{Var}[Q]}}{E[Q]} & =\sqrt{\alpha \cdot \left(\varepsilon -1\right)+\left(1-\alpha \right)\cdot \frac{s^{2}}{q^{2}}}, \end{array}$$

where the parameter ε must satisfy $\varepsilon >1-\frac{1-\alpha }{\alpha }\cdot \frac{s^{2}}{q^{2}}$ to ensure that the argument of the square root remains positive. The saturation limit (36) reduces to $1+\frac{s^{2}}{q^{2}}$ for instantaneous light pulses (α=0) and to unity for very long pulses (α=ε=1), leading to $\frac{\sqrt{N\cdot \mathrm{Var}[Q]}}{E[Q]}\to 0$. In the general case, f(x) is expected to saturate to a value depending on the dynamics of pixel recovery for the specific light pulse shape and duration, which is controlled by the parameter ε.

Examples of the function f(x) for different combinations of parameters are shown in the right-hand panel of Figure 2. Plotting f(x) instead of the scaled charge resolution $\frac{\sqrt{N\cdot \mathrm{Var}[Q]}}{E[Q]}$ allows the individual effects of the model parameters to be visualized more clearly, as indicated by the arrows. In the absence of correlated noise (γ=0), the function f(x) increases with *x* from the baseline value of $1+\frac{s^{2}}{q^{2}}$, reaches a maximum, and then decreases towards the saturation limit (36). For the limiting case of very long pulses (α=β=ε=1), this maximum occurs at x≈1.6, with only a weak dependence on s/q. This value corresponds to a mean of two avalanches per triggered pixel; since a primary avalanche always occurs when a pixel fires, the Poisson fluctuations arising from subsequent avalanches reach their maximum at this mean value. However, in a general situation, pixel recovery reduces the probability and mean charge of subsequent avalanches, causing a shift in the maximum of the function f(x), which is controlled by the parameter β.

Several fitting parameters are intrinsically correlated. In particular, γ and $\frac{s^{2}}{q^{2}}$ produce the same effect in the linear regime, whereas ε−1 and $\frac{s^{2}}{q^{2}}$ have a similar influence in the saturation limit, although their relative contributions depend on the value of α. In practice, the parameters γ and $\frac{s^{2}}{q^{2}}$ can be estimated independently from dark-count measurements (see Section 5.1), allowing the remaining parameters to be determined by fitting experimental data for $\frac{\sqrt{N\cdot \mathrm{Var}[Q]}}{E[Q]}$ or f(x). The recovery-related parameters α, β, and ε generally exhibit only weak correlations with the noise-related parameters γ and δ.

Finally, the proposed model can be readily extended to super-Poissonian light sources by including the corresponding contribution of seed fluctuations in the term $1+\frac{s^{2}}{q^{2}}$ in (33), as discussed in Appendix A.

### 2.4. Photon-Counting Resolution

In most applications, the measured SiPM signal is used to estimate either the number of photons impinging on the detector or a quantity proportional to it, such as the energy deposited in a scintillator. If the mean output charge is related to the mean number of incident photons through a known response function, $E\left[Q\right]=R(E\left[n_{\mathrm{ph}}\right])$, the reconstructed number of photons corresponding to a measured charge *Q* is given by $n_{\mathrm{ph}}^{*}\left(Q\right)=R^{-1}\left(Q\right)$. Consequently,

(38) $$n_{\mathrm{ph}}^{*}\left(E\left[Q\right]\right)=E\left[n_{\mathrm{ph}}\right]=\frac{N\cdot x}{PDE}\;.$$

The variance of the reconstructed number of photons, $\mathrm{Var}[n_{\mathrm{ph}}^{*}]$, is ultimately determined by the variance of the measured charge, Var[Q], which includes fluctuations associated with the photon statistics, avalanche multiplication, correlated noise, and nonlinear response. We define the scaled photon-counting resolution as

(39) $$\frac{\sqrt{N\cdot \mathrm{Var}[n_{\mathrm{ph}}^{*}]}}{E[n_{\mathrm{ph}}]}=\frac{E[Q]}{E[n_{\mathrm{ph}}]}\cdot {\left|\frac{dn_{\mathrm{ph}}^{*}}{dQ}\right|}_{E[Q]}\;\;\;\;\cdot \frac{\sqrt{N\cdot \mathrm{Var}[Q]}}{E[Q]}\;,$$

where, as in the scaled charge resolution $\frac{\sqrt{N\cdot \mathrm{Var}[Q]}}{E[Q]}$, the factor $\sqrt{N}$ is introduced to obtain a quantity independent of the total number of pixels.

For an ideal linear SiPM without correlated noise, the normalized mean charge $\frac{E[Q]}{N\cdot q}$ is given by (25), while $\frac{\sqrt{N\cdot \mathrm{Var}[Q]}}{E[Q]}$ is given by (26). In this case, (39) becomes

(40) $$\frac{\sqrt{N\cdot \mathrm{Var}[n_{\mathrm{ph}}^{*}]}}{E[n_{\mathrm{ph}}]}=\frac{\sqrt{N\cdot \mathrm{Var}[Q]}}{E[Q]}=\sqrt{\frac{1+\frac{s^{2}}{q^{2}}}{x}}\;.$$

For the limiting case of instantaneous light pulses, $\frac{E[Q]}{N\cdot q}$ and $\frac{\sqrt{N\cdot \mathrm{Var}[Q]}}{E[Q]}$ in the absence of correlated noise are given by (16) and (17), respectively, yielding

(41) $$\begin{array}{cc} \frac{\sqrt{N\cdot \mathrm{Var}[n_{\mathrm{ph}}^{*}]}}{E[n_{\mathrm{ph}}]} & =\frac{-1}{\ln\left(1-\frac{E[Q]}{N\cdot q}\right)}\cdot \frac{\frac{E[Q]}{N\cdot q}}{1-\frac{E[Q]}{N\cdot q}}\cdot \frac{\sqrt{N\cdot \mathrm{Var}[Q]}}{E[Q]}\\ \; & =\frac{1-e^{-x}}{x\cdot e^{-x}}\cdot \sqrt{\frac{1+\frac{s^{2}}{q^{2}}}{1-e^{-x}}-1}\;. \end{array}$$

As expected, this expression reduces to (40) in the limit x≪1. As *x* increases further, however, $\frac{\sqrt{N\cdot \mathrm{Var}[n_{\mathrm{ph}}^{*}]}}{E[n_{\mathrm{ph}}]}$ eventually diverges because $\frac{E[Q]}{N\cdot q}$ approaches its saturation value of unity (20). The scaled photon-counting resolution $\frac{\sqrt{N\cdot \mathrm{Var}[n_{\mathrm{ph}}^{*}]}}{E[n_{\mathrm{ph}}]}$ reaches a minimum at x≈1.5, with only a weak dependence on the ratio s/q. At this point, the mean fraction of fired pixels is already $\frac{E[Q]}{N\cdot q}=0.78$, indicating that the optimum operating point is achieved well within the nonlinear-response regime. This result highlights that nonlinear operation does not necessarily degrade the photon-counting performance. Instead, the reduction in the statistical fluctuations of the number of fired pixels more than compensates for the compression of the SiPM response.

**Figure 3:**
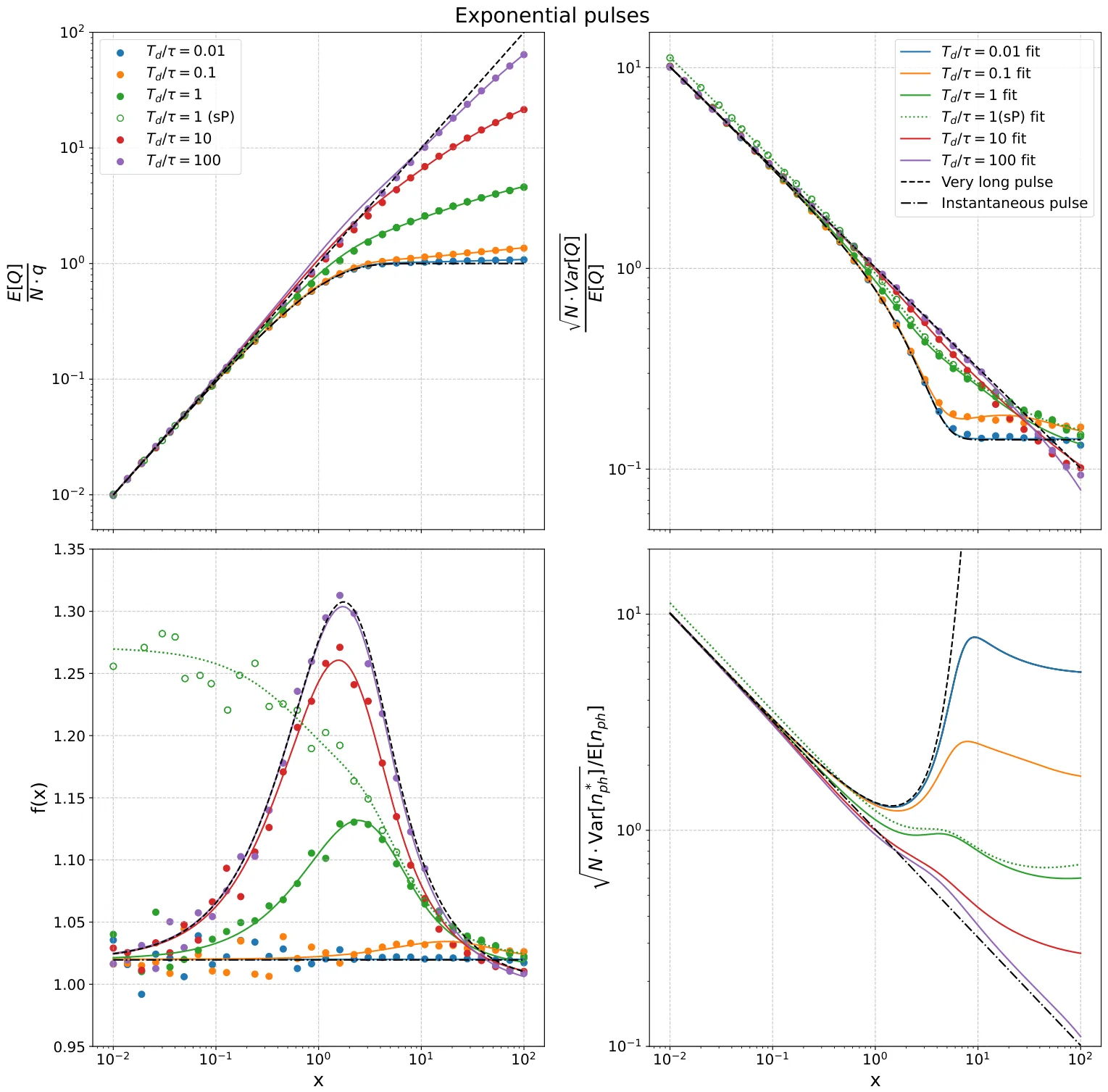
Simulation results for exponential light pulses in the absence of correlated noise. Fits of (27) to the normalized mean output charge $\frac{E[Q]}{N\cdot q}$ are shown in the **upper-left panel**. Fits of (30) and (33) to $\frac{\sqrt{N\cdot \mathrm{Var}[Q]}}{E[Q]}$ and f(x) for Poissonian light pulses are shown in the **upper-right** and **lower-left panels**, respectively. In addition, a representative simulation with super-Poissonian photon statistics is included, where f(x) is fitted using (A14). The corresponding scaled photon-counting resolution $\frac{\sqrt{N\cdot \mathrm{Var}[n_{\mathrm{ph}}^{*}]}}{E[n_{\mathrm{ph}}]}$ computed from (39) is shown in the **lower-right panel**. The black lines represent the exact analytical solutions for the limiting cases of instantaneous and very long light pulses.

**Figure 4:**
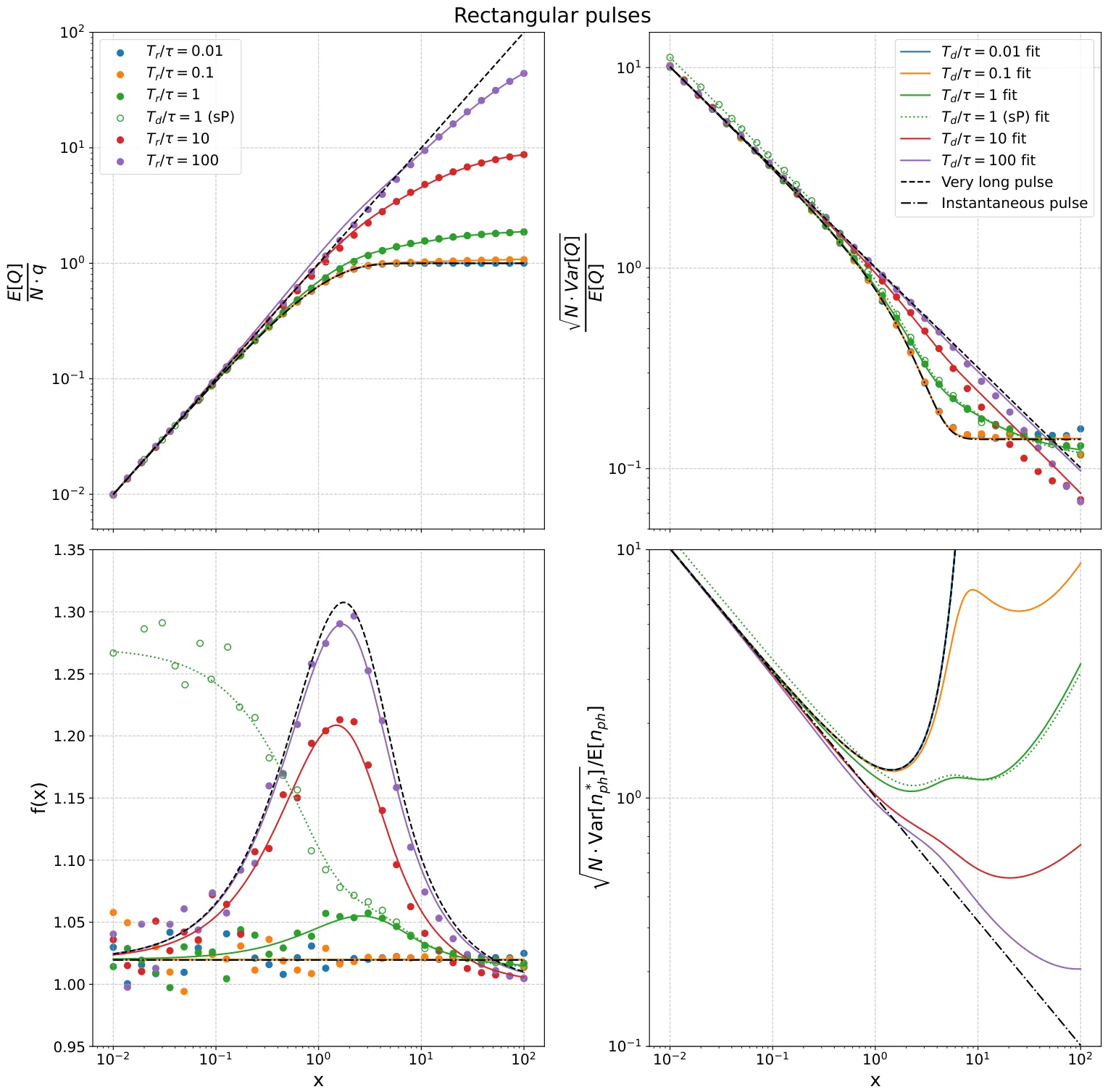
Same as Figure 3, but for rectangular light pulses. In this case, (28) is fitted to the normalized mean output charge in the **upper-left panel**.

**Figure 5:**
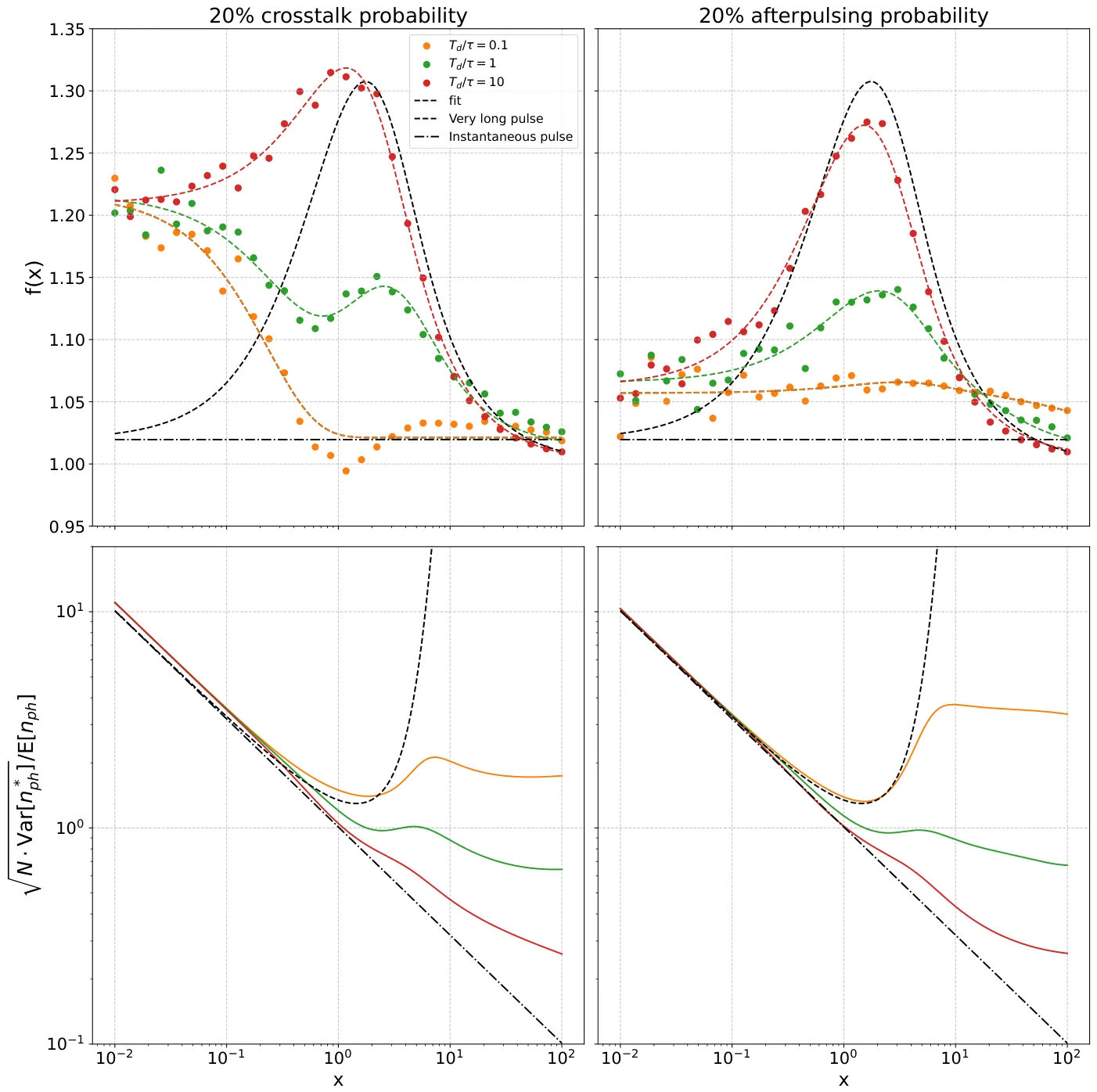
Simulation results for exponential light pulses assuming either a 20% crosstalk probability (**left**) or a 20% afterpulsing probability (**right**). Fits of (33) are shown in the upper panels. The corresponding scaled photon-counting resolution $\frac{\sqrt{N\cdot \mathrm{Var}[n_{\mathrm{ph}}^{*}]}}{E[n_{\mathrm{ph}}]}$ computed from (39) is shown in the lower panels. The black lines represent the exact analytical solutions for the limiting cases of instantaneous and very long light pulses in the absence of correlated noise.

For light pulses with durations comparable to the SiPM recovery time τ, including correlated noise, $\frac{E[Q]}{N\cdot q}$ can be modeled by either (27) or (28), depending on the pulse shape, while $\frac{\sqrt{N\cdot \mathrm{Var}[Q]}}{E[Q]}$ is described by (30) and (33). Once the model parameters have been determined from experimental or simulated data, $\frac{\sqrt{N\cdot \mathrm{Var}[n_{\mathrm{ph}}^{*}]}}{E[n_{\mathrm{ph}}]}$ follows directly from (39). Examples of $\frac{\sqrt{N\cdot \mathrm{Var}[n_{\mathrm{ph}}^{*}]}}{E[n_{\mathrm{ph}}]}$ for several simulated cases, together with the expressions (40) and (41) for the two limiting cases of very long and instantaneous light pulses, respectively, are shown in Figure 3, Figure 4 and Figure 5. For short pulses, $\frac{\sqrt{N\cdot \mathrm{Var}[n_{\mathrm{ph}}^{*}]}}{E[n_{\mathrm{ph}}]}$ also exhibits a minimum close to x=1.5, indicating that this feature is not restricted to the idealized limiting case of instantaneous pulses. The physical origin of this behavior is investigated in the following section using Monte Carlo simulations.

## 3. Validation with Simulation Data

The proposed fitting model (33) was validated using Monte Carlo simulations of the SiPM response. Simulations were performed for different light-pulse shapes and durations, both with and without correlated noise.

### 3.1. Simulation Code

The simulation code used in this work is based on the Monte Carlo framework presented in [32]. In a previous study [19], we introduced several improvements to its treatment of correlated noise, pixel recovery, and light-pulse generation. For the present work, the code was further extended to support both Poisson and negative binomial photon statistics, as well as stochastic avalanche-charge fluctuations, allowing the variance of the total output charge *Q* to be investigated.

For each simulation, the number of light pulses, the pulse shape, the photon statistics, and the SiPM characteristics (e.g., the number of pixels *N*, photon detection efficiency PDE, pixel recovery time τ, and correlated-noise probabilities) are specified. For every simulated pulse, the number of avalanche seeds $n_{s}$ and their arrival times are sampled according to the selected photon statistics, the PDE, and the temporal profile of the light pulse. The avalanche seeds are then randomly assigned to the *N* pixels and processed in chronological order.

The first seed reaching a given pixel always triggers an avalanche. Immediately after the avalanche, the pixel overvoltage $U_{p}$ drops to zero and subsequently recovers exponentially with time. Any later seed arriving at the same pixel can trigger a new avalanche with a probability that depends on the instantaneous value of $U_{p}$ [19]. When an avalanche is triggered, its mean charge $q(U_{p})$ is assumed to be proportional to the pixel overvoltage, while stochastic charge fluctuations are added by sampling from a normal distribution. The standard deviation of this distribution, $s(U_{p})$, was determined from single-photon charge spectra measured under dark conditions as a function of overvoltage (see Section 5.1). As an example, the corresponding ratio $\frac{s}{q}\left(U_{p}\right)$ for a Hamamatsu S13360-1350CS SiPM is shown in Figure 7. For the purpose of validating the fitting model (33), it is sufficient to specify the value of $\frac{s}{q}$ for fully recovered pixels together with a parameterization of $\frac{s}{q}\left(U_{p}\right)$ during pixel recovery. We found that this dependence is well approximated by an exponential function plus a constant, as illustrated in Figure 7.

Each avalanche may also generate correlated noise. Crosstalk is assumed to occur in the four nearest neighboring pixels [15,18], whereas afterpulses are generated in the same pixel after a random delay drawn from the characteristic afterpulse-time distribution of the SiPM (see [19] for details). The triggering probability and avalanche charge associated with these secondary avalanches depend on $U_{p}$ in the same way as for photon-induced avalanches. Furthermore, correlated-noise avalanches are allowed to generate additional correlated-noise events. Uncorrelated noise was neglected throughout the simulations.

The total output charge *Q* for each simulated pulse is obtained by summing the charge released by all avalanches generated during the event. The mean value E[Q] and the variance Var[Q] are then estimated from a sufficiently large ensemble of simulated pulses.

### 3.2. Simulation Results

We simulated the response of an SiPM to Poissonian light pulses with different shapes and durations. The results obtained for exponential and rectangular pulses are presented in Figure 3 and Figure 4, respectively. The curves are labeled according to the ratio of the pulse decay time $T_{d}$ to τ for exponential pulses, or by the ratio of the pulse duration $T_{r}$ to τ for rectangular pulses. For both pulse shapes, we also include a representative simulation of a super-Poissonian light source by assuming a negative binomial photon distribution with ζ=0.25 (see Appendix A), labeled as “sP”.

The normalized mean output charge $\frac{E[Q]}{N\cdot q}$ is shown as a function of the normalized input light intensity *x* in the upper-left panels, together with the corresponding least-squares fits of Equations (27) and (28). The scaled charge resolution $\frac{\sqrt{N\cdot \mathrm{Var}[Q]}}{E[Q]}$ and the function f(x) are displayed in the upper-right and lower-left panels, respectively, together with the corresponding fits. Because the details of the different contributions to charge fluctuations are more pronounced in f(x), the least-squares fits of Equation (33) were first performed on the simulated data of f(x). The fitted curves for $\frac{\sqrt{N\cdot \mathrm{Var}[Q]}}{E[Q]}$ were then obtained directly from f(x) using Equation (30). The corresponding scaled photon-counting resolution $\frac{\sqrt{N\cdot \mathrm{Var}[n_{\mathrm{ph}}^{*}]}}{E[n_{\mathrm{ph}}]}$ computed from (39) is shown in the lower-right panels. The functions $\frac{\sqrt{N\cdot \mathrm{Var}[n_{\mathrm{ph}}^{*}]}}{E[n_{\mathrm{ph}}]}$ for the limiting cases of very long pulses (40) and instantaneous pulses (41) are also shown for comparison. Since all quantities are properly normalized, the results are independent of the simulation parameters *N*, PDE, and τ.

Correlated noise was not included in these simulations, so the fitting parameters were fixed to c=d=γ=δ=0. We assumed $\frac{s}{q}=0.15$, a deliberately large value selected to better illustrate the saturation of $\frac{\sqrt{N\cdot \mathrm{Var}[Q]}}{E[Q]}$ for short light pulses predicted by (21).

As expected, the SiPM response is nearly linear for very long light pulses, whereas it approaches the instantaneous-pulse limit (14) as the pulse duration becomes much shorter than the pixel recovery time. For intermediate pulse durations comparable to τ, both (27) and (28) accurately reproduce the simulated mean output charge. The fitted values of the parameters *a* and *b* are listed in Table 1. Their statistical uncertainties are generally below 5%, except for very short pulses, where a≈0 and b≈0, reflecting the negligible effect of pixel recovery. Since $\frac{E[Q]}{N\cdot q}$ depends only on the first moment of the photon-number distribution, the fitted values of *a* and *b* are nearly identical for Poissonian and super-Poissonian light pulses with the same temporal profile.

Regarding the output-charge fluctuations, the functions $\frac{\sqrt{N\cdot \mathrm{Var}[Q]}}{E[Q]}$ and f(x) are accurately described by (30) and (33) (or (A14) for the representative super-Poissonian pulse), respectively, with fit residuals below 3% for f(x). The fitted values of the parameters α, β, and ε are listed in Table 1. For the super-Poissonian pulse, the parameter ζ was fixed to 0.25, leading to a significant increase in the charge fluctuations at small *x* values. Nevertheless, the remaining fitting parameters are almost identical to those obtained for Poissonian pulses with the same duration.

The parameter α decreases as the pulse duration becomes shorter. For very short pulses, α=0, yielding $f\left(x\right)=1+\frac{s^{2}}{q^{2}}$ for Poissonian photon statistics. Consequently, $\frac{\sqrt{N\cdot \mathrm{Var}[Q]}}{E[Q]}$ reduces to (17). The behavior of the parameters β and ε is less straightforward. In general, however, β decreases with decreasing pulse duration, shifting the maximum of f(x) toward larger values of *x*. The shorter the light pulse, the higher the value of *x* to reach a mean of two avalanches per triggered pixel. The parameter ε remains close to unity in all simulated cases. In fact, variations in ε become appreciable only for x≳30 and long pulses with α>0.1, indicating that ε=1 can be safely assumed whenever the analysis is restricted to smaller *x* values. For instance, ε=1.14±0.11 and ε=1.019±0.012 were obtained for the cases of exponential pulses with $T_{d}/\tau =0.1$ and $T_{d}/\tau =1$, respectively. As for the parameters *a* and *b*, the uncertainties in α and β are generally below 5%. They become significantly larger only for short pulses where α≈0 and (33) becomes only weakly sensitive to relative variations in α and β.

For both pulse shapes with Poissonian photon statistics, the scaled photon-counting resolution $\frac{\sqrt{N\cdot \mathrm{Var}[n_{\mathrm{ph}}^{*}]}}{E[n_{\mathrm{ph}}]}$ approaches (40) and (41) in the limits of very long and very short light pulses, respectively. For the representative super-Poissonian pulse, $\frac{\sqrt{N\cdot \mathrm{Var}[n_{\mathrm{ph}}^{*}]}}{E[n_{\mathrm{ph}}]}$ is slightly larger because of the additional excess-noise contribution parameterized by ζ in (A14), although its overall behavior remains very similar to that of the Poissonian case.

For short light pulses (i.e., either $T_{d}/\tau \leq 1$ or $T_{r}/\tau \leq 1$), $\frac{\sqrt{N\cdot \mathrm{Var}[n_{\mathrm{ph}}^{*}]}}{E[n_{\mathrm{ph}}]}$ exhibits a minimum at x≈1.5. In contrast, for long light pulses (i.e., either $T_{d}/\tau >1$ or $T_{r}/\tau >1$), the minimum shifts toward larger values of *x*, and in some cases disappears altogether, yielding a monotonically decreasing function. The behavior also differs between exponential and rectangular pulses because recovering pixels contribute more significantly to the total output charge in the exponential case. For short rectangular pulses, $\frac{E[Q]}{N\cdot q}$ saturates as x→∞ according to (28), causing the factor $\frac{dn_{\mathrm{ph}}^{*}}{dQ}$ in (39) to diverge and, consequently, $\frac{\sqrt{N\cdot \mathrm{Var}[n_{\mathrm{ph}}^{*}]}}{E[n_{\mathrm{ph}}]}$ also diverges. By contrast, for short exponential pulses, $\frac{E[Q]}{N\cdot q}$ continues to increase slowly even at very large *x* values according to (27). Therefore, $\frac{dn_{\mathrm{ph}}^{*}}{dQ}$ remains finite and $\frac{\sqrt{N\cdot \mathrm{Var}[n_{\mathrm{ph}}^{*}]}}{E[n_{\mathrm{ph}}]}$ decreases for x≳10.

Simulation results for an SiPM including correlated noise are shown in Figure 5 for exponential light pulses with different values of $T_{d}/\tau$. The upper panels show the simulated f(x) together with the corresponding least-squares fits of (33), including the parameters γ and δ that account for correlated noise. A crosstalk probability of 20% was assumed for the left-hand panels, whereas an afterpulsing probability of 20% was assumed for the right-hand panels. These probabilities were intentionally chosen to be larger than those typically observed in commercial SiPMs in order to illustrate more clearly the impact of correlated noise on f(x). As in the previous simulations, $\frac{s}{q}=0.15$ was assumed. The corresponding scaled photon-counting resolution $\frac{\sqrt{N\cdot \mathrm{Var}[n_{\mathrm{ph}}^{*}]}}{E[n_{\mathrm{ph}}]}$, computed from (39), is displayed in the lower panels. Again, the curves of $\frac{\sqrt{N\cdot \mathrm{Var}[n_{\mathrm{ph}}^{*}]}}{E[n_{\mathrm{ph}}]}$ for the limiting cases of very long pulses (40) and instantaneous pulses (41) are also shown for comparison. The functions $\frac{E[Q]}{N\cdot q}$ and $\frac{\sqrt{N\cdot \mathrm{Var}[Q]}}{E[Q]}$ are not shown because they differ only slightly from those in Figure 3 obtained without correlated noise for the same values of $T_{d}/\tau$.

The model (27) for $\frac{E[Q]}{N\cdot q}$ and the model (33) for f(x) also provide excellent fits to the simulation data including correlated noise, with residuals below 3%. The fitted values of the parameters *a*, *b*, α, β, and ε are very similar to those listed in Table 1 for the corresponding values of $T_{d}/\tau$. The remaining fitting parameters *c*, *d*, γ, and δ, which account for correlated noise, are listed in Table 2.

For the simulations including crosstalk, the fitted values of both *c* and γ are close to the imposed crosstalk probability of 0.20, consistent with the theoretical predictions for the excess mean charge and excess noise factor due to crosstalk reported in [17,18]. The parameters *d* and δ increase as the pulse duration decreases. Since crosstalk occurs on timescales much shorter than the pixel recovery time, its contribution is rapidly suppressed as *x* increases for short light pulses. Moreover, crosstalk may occupy neighboring pixels before subsequent photons arrive, thereby reducing the probability that these photons trigger additional avalanches. As a consequence, f(x) falls below unity for short light pulses and values of *x* around 1. This subtle effect is not reproduced by model (33). Although the parameters *c* and γ are relatively small, their uncertainties remain below approximately 5%, since the fits are highly sensitive to these parameters in the limit x→0. In contrast, the uncertainties in *d* and δ are typically larger (≳10%), reflecting the comparatively small effect of crosstalk suppression relative to that of pixel recovery.

Interestingly, the behavior of f(x) in the presence of crosstalk resembles that obtained for the representative super-Poissonian pulse shown in Figure 3. In both cases, f(x) can be modeled by adding a term that decreases with increasing *x*.

An afterpulsing probability of 20% yields comparatively small values of c=0.079 and γ=0.041 because afterpulses are typically generated while the pixel is still only partially recovered, resulting in a lower average avalanche charge and smaller charge fluctuations than those produced by avalanches in fully recovered pixels. Unlike crosstalk, afterpulsing occurs after a finite delay in the same recovering pixel and is therefore not significantly suppressed as *x* increases for very short light pulses. For long light pulses, the combined contributions of impinging photons and afterpulses approach the saturation limit given by (37) only gradually. Consequently, we fixed d=δ=0. The relative uncertainties in *c* and γ are typically larger than for crosstalk because the corresponding contributions of afterpulsing to the SiPM response are significantly smaller.

## 4. Comparison with Previous Model

We compared the predictions of the photon-counting resolution from our proposed model with those from the model of S. Vinogradov et al. [21,31], which is the only previous analytical model applicable for finite-duration light pulses of arbitrary intensity, as far as we know.

In Ref. [21], two distinct scenarios were considered: light pulses of duration much less than the pixel recovery time τ, and pulses of duration greater than τ. The first scenario was modeled using a binomial distribution for the number of triggered pixels. In this case, $\frac{E[Q]}{N\cdot q}$ is given by (16) in the absence of correlated noise. For the second scenario of long light pulses, the SiPM was modeled as *N* independent Geiger counters with a non-paralyzable dead time $\tau_{d}$. Assuming rectangular pulses of duration $T_{r}\gg \tau_{d}$, the normalized mean output charge in the absence of correlated noise is

(42) $$\frac{E[Q]}{N\cdot q}=\frac{x}{1+x\cdot \frac{\tau_{d}}{T_{r}}}.$$

Although $\tau_{d}$ was identified with the pixel recovery time τ in [21], we chose to make the model more flexible by treating $\tau_{d}$ as a free fitting parameter. This provides the previous model with the best possible baseline for comparison, as $\tau_{d}$ can be interpreted as an effective dead time that approximately accounts for the actual dynamics of pixel recovery, as described in [24].

**Figure 6:**
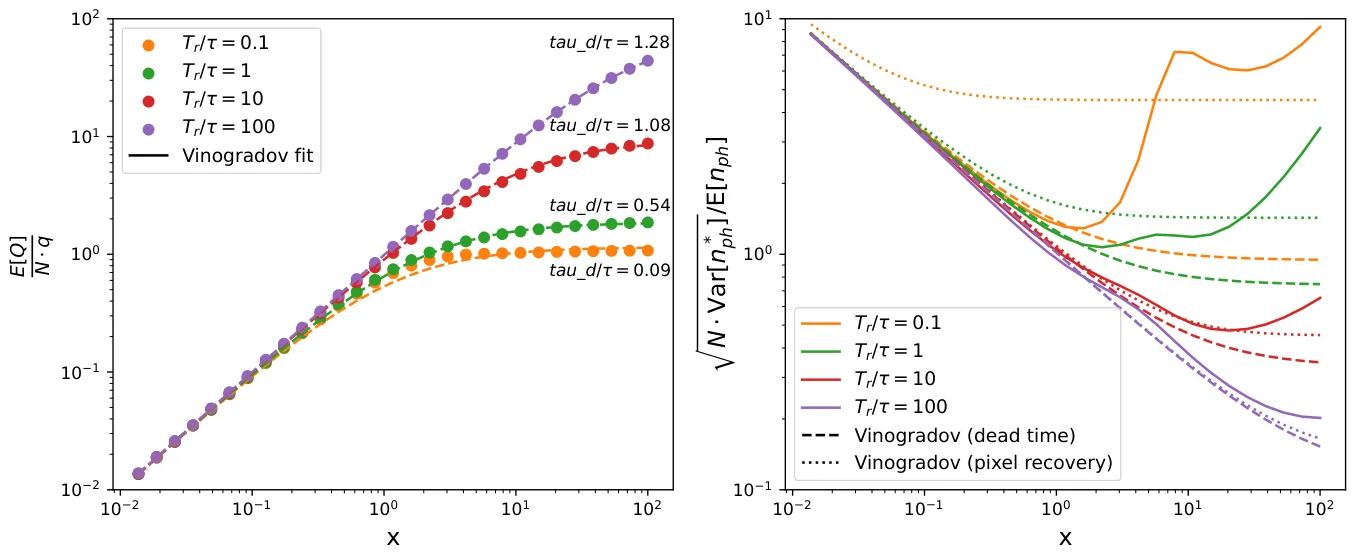
(**Left-hand panel**): fits of the dead-time model (42) to the same simulated data for rectangular pulses in the absence of correlated noise shown in Figure 4. (**Right-hand panel**): predictions of the models proposed in [21,31] compared with the predictions from our proposed model for the same simulated cases shown in the **left-hand panel**.

In the left-hand panel of Figure 6, least-square fits of expression (42) are shown for the same simulated data of Poissonian rectangular pulses with $\frac{T_{r}}{\tau }\geq 0.1$ previously presented in Figure 4. The ratio of the effective dead time to the pixel recovery time $\frac{\tau_{d}}{\tau }$ is indicated near each curve. Expression (42) describes the simulated mean output charge data as accurately as our model (28) for long pulses. However, even with the flexibility granted by treating $\tau_{d}$ as a free fitting parameter, this model fails to properly reproduce simulated data for $\frac{T_{r}}{\tau }<1$, because the non-paralyzable dead-time framework is no longer valid in that regime. Furthermore, expression (42) is not valid for exponential-like pulses, where $\frac{E[Q]}{N\cdot q}$ grows logarithmically with *x* in the limit x→∞, as shown in Figure 3.

In [21], the photon-counting resolution is described as

(43) $$\frac{\sqrt{\mathrm{Var}[n_{\mathrm{ph}}^{*}]}}{E[n_{\mathrm{ph}}]}=\sqrt{\frac{ENF}{E[n_{\mathrm{ph}}]}},$$

where ENF is the excess noise factor with respect to the input Poisson photon statistics, which results from the product of the partial excess noise factors $F_{i}$ of all the stochastic processes that influence the SiPM response. This approach assumes that the output of each stochastic process approximately preserves the Poisson statistics (i.e., Fano factor equal to unity), which is not true in general. To compare with our model, we only consider here the reported partial excess noise factors associated with the photon detection efficiency $F_{PDE}=\frac{1}{PDE}$, the avalanche multiplication $F_{m}=1+\frac{s^{2}}{q^{2}}$, and the nonlinear response $F_{\mathrm{nl}}$. This last factor is

(44) $$F_{\mathrm{nl}}=\frac{e^{x}-1}{x}$$

for very short light pulses, and

(45) $$F_{\mathrm{nl}}=1+x\cdot \frac{\tau_{d}}{T_{r}}.$$

for long pulses.

In Ref. [31], the above model was modified by estimating a factor $F_{\mathrm{nl}}$ that takes into account the distribution of arrival times of photons and the pixel recovery function. Under certain simplifying assumptions, the following analytical expression was obtained

(46) $$F_{\mathrm{nl}}=\frac{{\left(1+x\cdot \frac{\tau }{T_{r}}\right)}^{2}}{1+\frac{x}{2}\cdot \frac{\tau }{T_{r}}}.$$

In the right-hand panel of Figure 6, for the same simulated cases shown in the left-hand panel, the predictions for the scaled photon-counting resolution $\frac{\sqrt{N\cdot \mathrm{Var}[n_{\mathrm{ph}}^{*}]}}{E[n_{\mathrm{ph}}]}$ from the models presented in [21,31] are compared with those from our model. Similar results are obtained for very long rectangular pulses with $\frac{T_{r}}{\tau }=100$, but the models proposed in [21,31] fail to describe $\frac{\sqrt{N\cdot \mathrm{Var}[n_{\mathrm{ph}}^{*}]}}{E[n_{\mathrm{ph}}]}$ for shorter pulses. Remarkably, these models predict that $\frac{\sqrt{N\cdot \mathrm{Var}[n_{\mathrm{ph}}^{*}]}}{E[n_{\mathrm{ph}}]}$ asymptotically approaches a constant value in the limit x→∞, which is inconsistent with the fact that the SiPM response saturates in this limit for rectangular pulses. This discrepancy arises directly from assuming that the total ENF is the product of the partial excess noise factors $F_{i}$. To illustrate this breakdown, we compare $\frac{\sqrt{N\cdot \mathrm{Var}[n_{\mathrm{ph}}^{*}]}}{E[n_{\mathrm{ph}}]}$ for the limiting case of very short pulses obtained with this approach,

(47) $$\frac{\sqrt{N\cdot \mathrm{Var}[n_{\mathrm{ph}}^{*}]}}{E[n_{\mathrm{ph}}]}=\frac{\sqrt{\left(e^{x}-1\right)\cdot \left(1+\frac{s^{2}}{q^{2}}\right)}}{x},$$

with the exact solution (41). Both expressions only coincide if s=0 (i.e., avalanche multiplication introduces no excess noise). Otherwise, expression (47) diverges as $\frac{\sqrt{e^{x}\cdot \left(1+\frac{s^{2}}{q^{2}}\right)}}{x}$, while expression (41) diverges much faster as $\frac{e^{x}}{x}\cdot \frac{s}{q}$.

## 5. Experimental Validation

We experimentally validated the proposed fitting models using three different light sources and two Hamamatsu SiPMs. A scintillation crystal, a nanosecond laser, and a pulsed LED were employed to provide light pulses with different temporal characteristics. The SiPM models S13360-1325CS (N=2668) and S13360-1350CS (N=667) were evaluated. First, the characterization measurements performed under dark conditions are described, followed by the experimental procedures used to measure the SiPM response to scintillation, laser, and LED pulses. Finally, the measured response and photon-counting resolution in the nonlinear regime are compared with the proposed models.

### 5.1. Characterization Measurements Under Dark Conditions

Charge spectra of dark count events were measured for both SiPM models, S13360-1325CS and S13360-1350CS. During these measurements, the SiPM was placed inside a light-tight box and biased using a Hamamatsu C12332 driver circuit, which includes an operational amplifier and a compensation system for the temperature dependence of the SiPM gain. The output signal was digitized with a Tektronix TDS5032B digital oscilloscope with a bandwidth of 350 MHz. The oscilloscope was configured to trigger on single-avalanche signals and integrate the corresponding waveforms. The resulting integral, proportional to the collected charge, was expressed in units of nV·s. The integration window was chosen to be sufficiently long to include afterpulses.

**Figure 7:**
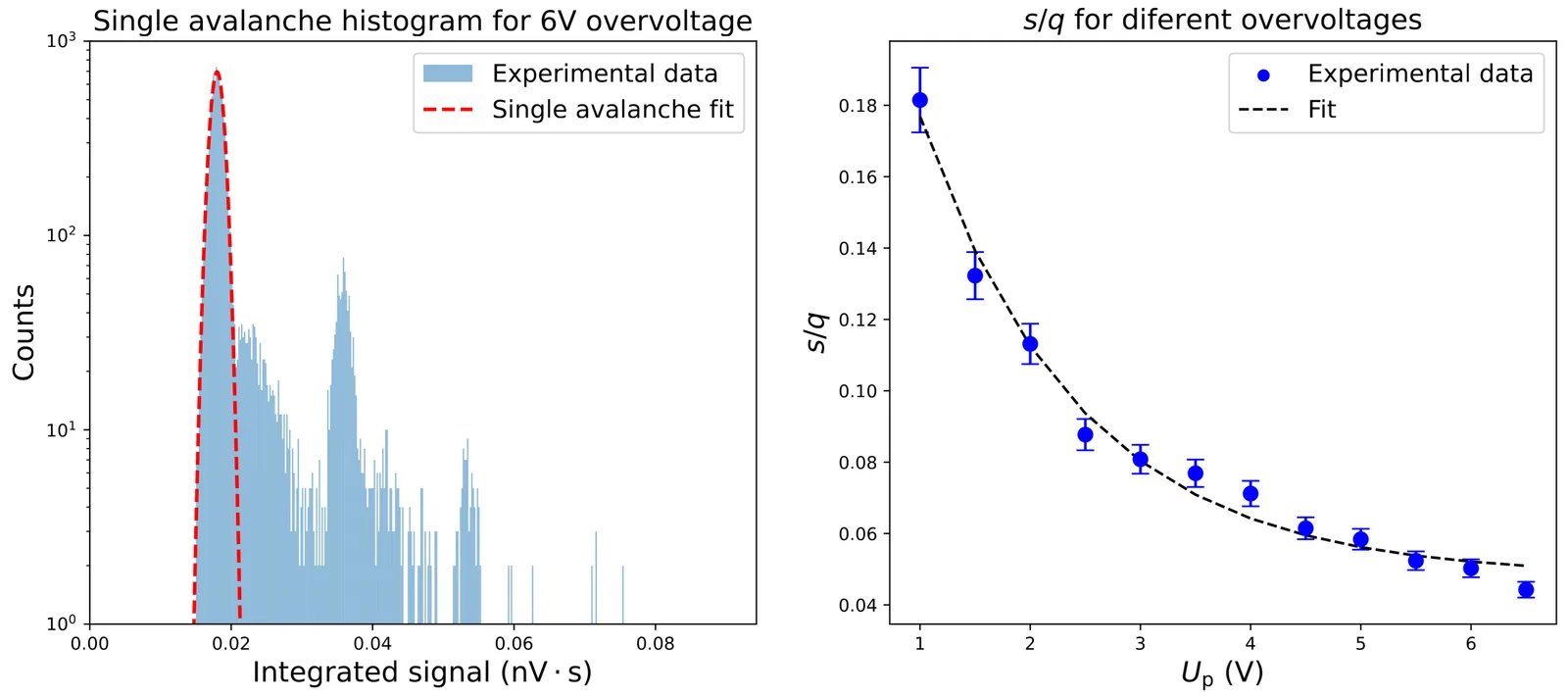
Measurements of the output charge of a Hamamatsu S13360-1350CS SiPM under dark conditions. The **left-hand panel** shows the charge histogram of dark count events measured at an overvoltage of 6 V. The primary peak, corresponding to single-avalanche events, is fitted with a Gaussian distribution. The **right-hand panel** shows the experimental ratio of the standard deviation *s* to the mean avalanche charge *q* of this peak as a function of overvoltage. The dashed line represents a fit to an exponential function plus a constant.

The charge histogram was then constructed after subtracting the pedestal, which was determined from randomly acquired waveforms without avalanche signals. The pedestal fluctuations were negligible compared to the single-avalanche charge dispersion. An example of a charge histogram measured with the S13360-1350CS SiPM at an overvoltage of 6 V is shown in the left-hand panel of Figure 7.

The charge histogram provides several useful characteristics of the SiPM response. The first peak corresponds to single-avalanche events without correlated noise. As illustrated in Figure 7, this peak was fitted with a Gaussian function to determine the mean avalanche charge *q* and its standard deviation *s*. The resulting ratio $\frac{s}{q}$ as a function of overvoltage for the S13360-1350CS SiPM is shown in the right-hand panel of Figure 7. The remaining peaks correspond to events with crosstalk, whereas the counts accumulated between peaks are due to afterpulsing. Consequently, the crosstalk and afterpulsing probabilities can be determined directly from the fractions of events identified as each type of correlated-noise event.

More importantly, the parameters *c* and γ can also be determined directly from the first two moments of the charge distribution. Neglecting the small probability that two or more dark count events occur within the integration window, these measurements can be described using the theoretical framework of Section 2 with $E\left[n_{f}\right]=E\left[n_{s}\right]=1$ and $\mathrm{Var}\left[n_{f}\right]=\mathrm{Var}\left[n_{s}\right]=0$. Under these conditions, the dimensionless parameter takes the fixed value $x=\frac{1}{N}\ll 1$, and expressions (29) and (35) reduce to

(48) $$\begin{array}{cc} E[Q] & =q\cdot (1+c)\;, \end{array}$$

(49) $$\begin{array}{cc} \mathrm{Var}[Q] & =\left(s^{2}+\gamma \cdot q^{2}\right)\cdot {\left(1+c\right)}^{2}\;. \end{array}$$

Since these SiPMs exhibit relatively low levels of correlated noise, the statistical uncertainties in *c* and γ are comparatively large, ranging from approximately 10% to 40%. The resulting values of $\frac{s}{q}$, *c*, and γ for both SiPMs are listed in Table 4. It should be emphasized that the parameters *c* and γ effectively describe the mean and variance contributions from correlated noise, incorporating noise cascades associated with the specific pixel geometry.

### 5.2. Experimental Procedure Using a Scintillation Crystal

The experimental arrangement employing a scintillation crystal follows the methodology detailed in [24]. A 3×3×20 mm^3^ LYSO(Ce) scintillator (light yield of 29 ph/keV and decay time of 42 ns), wrapped with a BaSO_4_ reflector, was optically coupled to a Hamamatsu S13360-1350CS SiPM (N=667 and τ=29 ns) using silicone grease. The scintillator was irradiated using the X-ray and γ-ray emitting isotopes listed in Table 3. The SiPM was biased using a Hamamatsu C12332 driver circuit without additional amplification. The complete setup was enclosed in a light-tight box.

**Figure 8:**
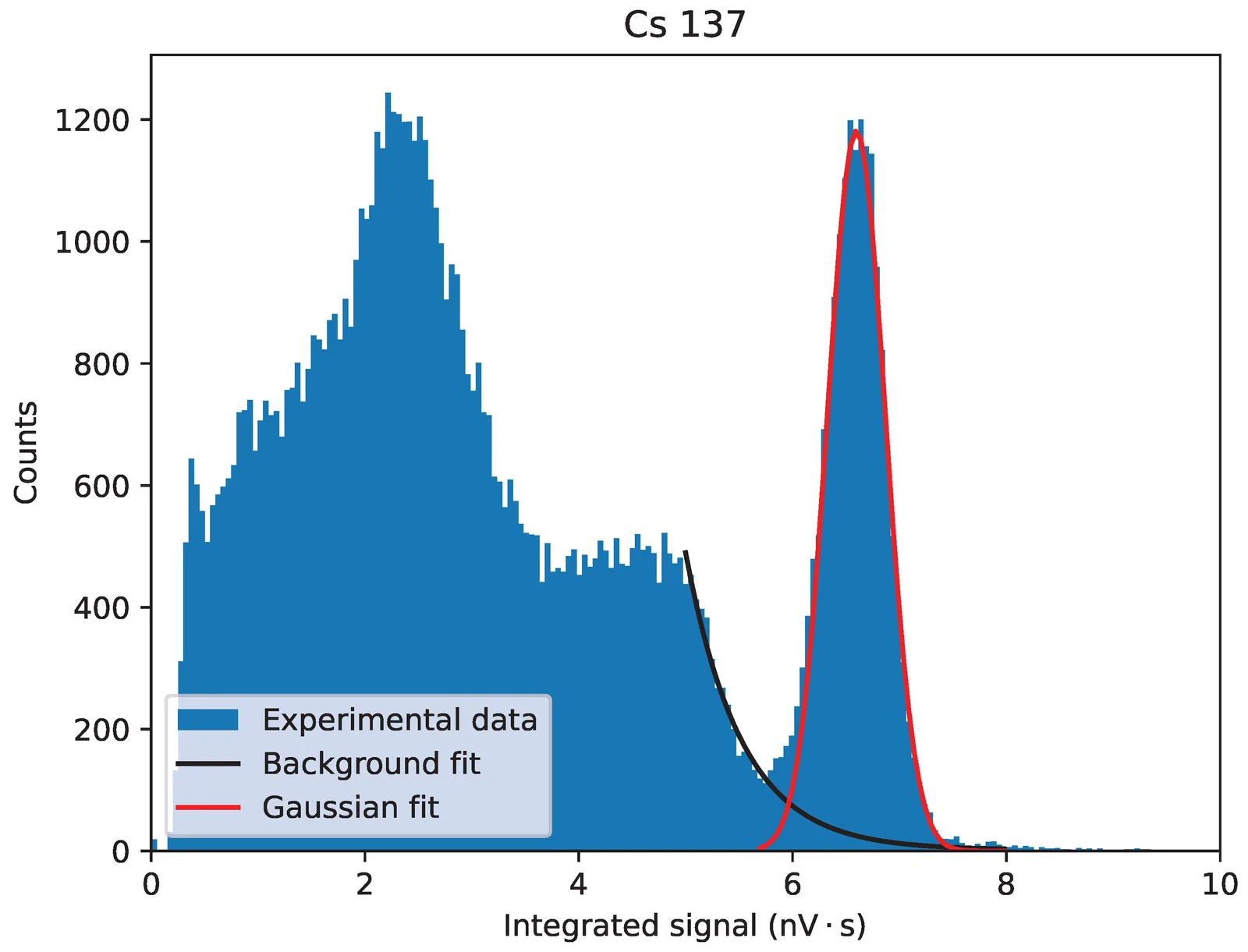
Charge histogram measured with a Hamamatsu S13360-1350CS SiPM coupled to a LYSO scintillator and irradiated with a ^137^Cs γ-ray source. The 662 keV photopeak is fitted with a Gaussian function after background subtraction. The underlying background is modeled by an exponential function plus a constant.

The output charge was corrected for the pedestal and histogrammed using the digital oscilloscope. The identified photopeaks were then fitted with Gaussian functions to determine their mean charge and variance. Before each fit, the underlying background was subtracted from each photopeak. As an example, Figure 8 shows the charge spectrum measured for a ^137^Cs γ-ray source, together with the Gaussian fit to the 662 keV photopeak and the corresponding background model, which is dominated by the Compton edge. For lower-energy photopeaks, the intrinsic background of the LYSO scintillator must also be taken into account. In all cases, the background in the vicinity of each photopeak was well described by an exponential function plus a constant.

To validate the proposed model, the photopeak energies, $E_{\gamma }$, were converted into the corresponding mean number of avalanche seeds per pixel *x*. For this purpose, the photopeaks in the energy interval from 31 to 122 keV, where the SiPM response remains linear, were fitted with

(50) $$\frac{E[Q]}{N\cdot q}=\left(1+c\right)\cdot k\cdot E_{\gamma }\;,$$

where *q* and *c* were obtained from the characterization measurements under dark conditions described in Section 5.1. The fitting parameter *k* defines the conversion factor between $E_{\gamma }$ and *x*, namely $x=k\cdot E_{\gamma }$. This parameter incorporates the PDE, the scintillation light yield, and the optical light-collection efficiency. For the S13360-1350CS SiPM operated at an overvoltage of 4 V, we obtained $k=1.38\cdot 10^{-3}$ keV^−1^, in agreement with the value reported in [24].

Because the scintillator length is much larger than its transverse dimensions, the light-collection efficiency depends on the interaction position of the γ ray within the crystal. Consequently, the number of photons reaching the SiPM for a given photopeak exhibits super-Poissonian fluctuations, resulting in an additional broadening of the measured photopeaks.

### 5.3. Experimental Procedure Using a Laser or an LED

**Figure 9:**
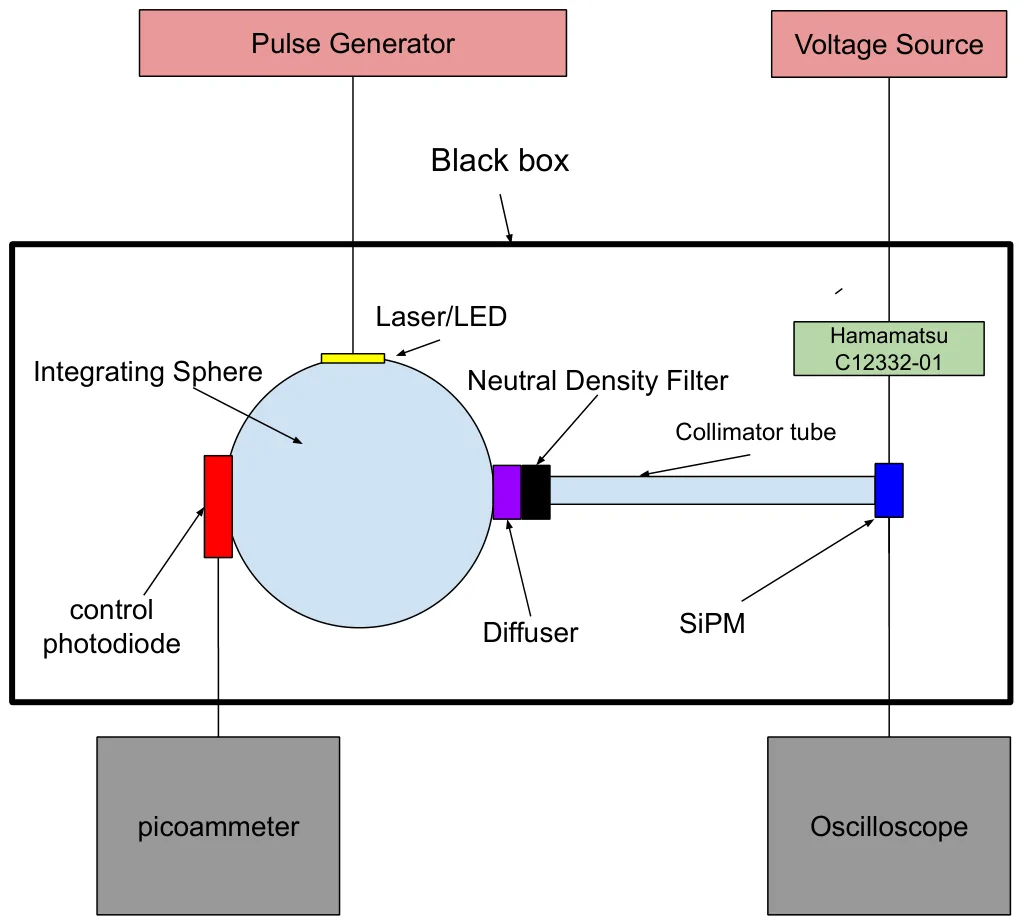
Schematic diagram of the experimental setup used for the measurements with laser and LED pulses.

A second experimental setup was employed to characterize the SiPM response using either a PicoQuant LDH 8-1-1283 laser (650 nm wavelength, 100 ps pulse width), driven by a PicoQuant PDL 800-B pulsed laser driver, or a Kingbright L7113QBCD pulsed LED (465 nm wavelength), driven by an Agilent 33522A waveform generator. A schematic diagram of this setup is shown in Figure 9. The setup comprised an integrating sphere mounted on an optical bench inside a light-tight box. The light source was coupled to one of the sphere ports, while a Thorlabs FDS1010 photodiode, connected to a picoammeter, was placed at a perpendicular port to monitor the input light intensity $I_{\mathrm{sph}}$. The SiPM was placed at a third port. To ensure uniform illumination of the SiPM, a diffuser was used. The intensity inside the sphere $I_{\mathrm{sph}}$ was adjusted by varying the light-source intensity. The light intensity reaching the SiPM $I_{\mathrm{SiPM}}$ was further attenuated using neutral-density filters with different optical densities together with collimator tubes of different lengths placed between the integrating sphere and the SiPM. This arrangement allowed measurements to be performed over a wide range of incident light intensities. For these measurements, we used both SiPM models S13360-1325CS and S13360-1350CS. As in the previous measurements, the SiPMs were biased by the Hamamatsu C12332 driver circuit and the output signal was digitized by the oscilloscope, which was triggered by a signal synchronized with the light source. We combined measurements with and without the amplification stage of the driver circuit, depending on the input light intensity. The recorded waveforms were integrated to obtain the mean E[Q] and variance Var[Q].

**Figure 10:**
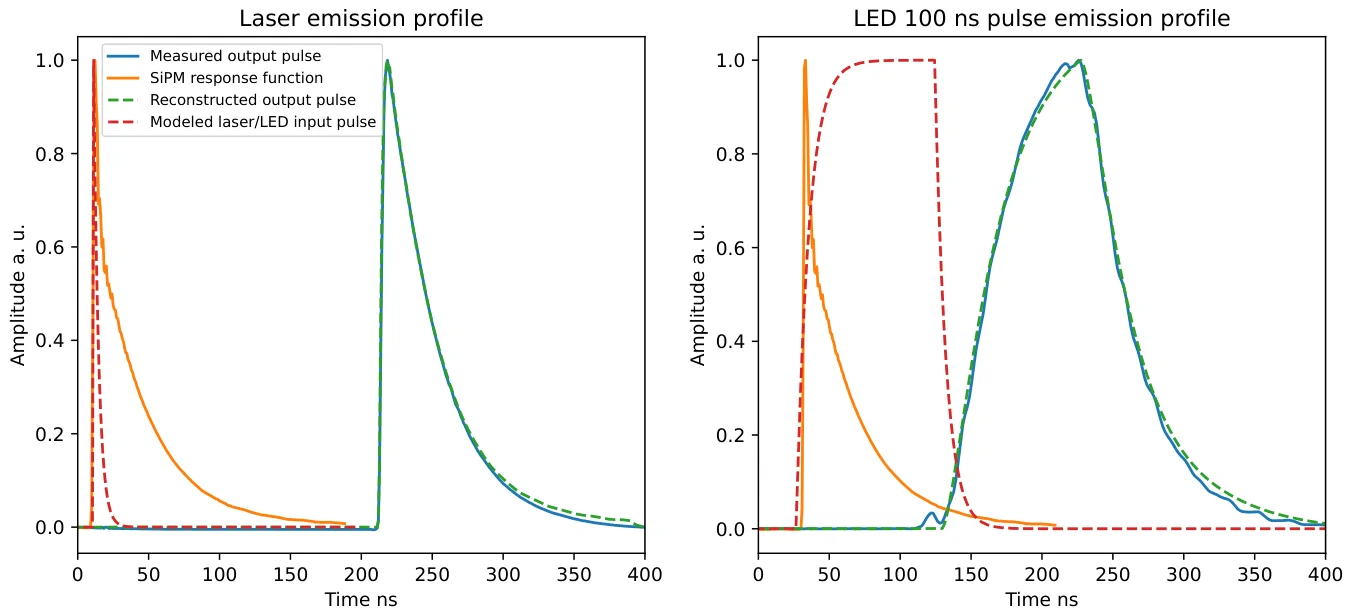
Normalized average waveforms measured for laser pulses (**left**) and 100 ns rectangular LED pulses (**right**). The input light profiles were reconstructed taking into account the average SiPM response function (see text for details).

Examples of normalized average waveforms measured for laser pulses and 100ns rectangular LED pulses with the S13360-1350CS SiPM model are shown in Figure 10. These waveforms were acquired using the amplification stage at low light intensity to avoid distortion caused by SiPM non-linearity. Nevertheless, they exhibit noticeable broadening due to the finite response function of the SiPM, particularly when using the amplifier stage. The figure also shows the reconstructed input light profiles from the measured average waveforms for light pulses and for dark counts. The latter provides the SiPM response function for a single photon, including correlated noise. The input light profile for laser pulses was modeled by an exponential decay function, leaving the decay time as a free fitting parameter. The convolution of this function with the SiPM response function is then least-squares fitted to the output waveform for laser pulses. This method yields a laser decay time of 3.5 ns. For the LED pulses, the input light profile was modeled as a charge-discharge pulse, where the rise and decay times are fitting parameters. The least-squares fit of the convolution to the output waveform yields values of 9 ns and 8 ns for the rise and decay times, respectively.

The input light intensity $I_{\mathrm{sph}}$ was converted into the mean number of avalanche seeds per pixel *x* following the same procedure used for the scintillator crystal. Specifically, the conversion factor was obtained from measurements of the mean output charge E[Q] in the linear region, together with the parameters *q* and *c* determined under dark conditions. In this case, the calibration constant implicitly accounts for the attenuation factor between $I_{\mathrm{sph}}$ and $I_{\mathrm{SiPM}}$, which arises from the neutral-density filters and collimator tubes. Notably, this method does not require $I_{\mathrm{sph}}$ to be measured in absolute units. This procedure is applicable only when the attenuation factor is sufficiently large, such that $I_{\mathrm{sph}}$ remains above the photodiode sensitivity threshold while $I_{\mathrm{SiPM}}$ is low enough for the SiPM to operate in its linear range.

As an independent validation, we also used an alternative procedure to convert $I_{\mathrm{sph}}$ into *x* based on a second Thorlabs FDS1010 photodiode with NIST-traceable calibration (5% uncertainty). In each setup configuration, the SiPM was replaced by the calibrated photodiode to measure $I_{\mathrm{SiPM}}$ in absolute units. Using the ratio of the active areas of the SiPM and the photodiode, we determined the conversion factor from $I_{\mathrm{sph}}$ to the mean number of photons impinging on the SiPM $E[n_{\mathrm{ph}}]$. To convert $E[n_{\mathrm{ph}}]$ to *x*, the PDE at the wavelength of the light source is needed. To determine the PDE, we used the same setup with the SiPM, using a sufficiently large attenuation factor to ensure that $E[n_{\mathrm{ph}}]∼1$. Under these conditions, the probability of observing no SiPM signal is $e^{-PDE\cdot E[n_{\mathrm{ph}}]}$, according to Poisson statistics. The estimated uncertainty of the resulting PDE values is approximately 10%. Both calibration procedures yielded consistent values of *x* within their respective uncertainties.

### 5.4. Experimental Results

**Figure 11:**
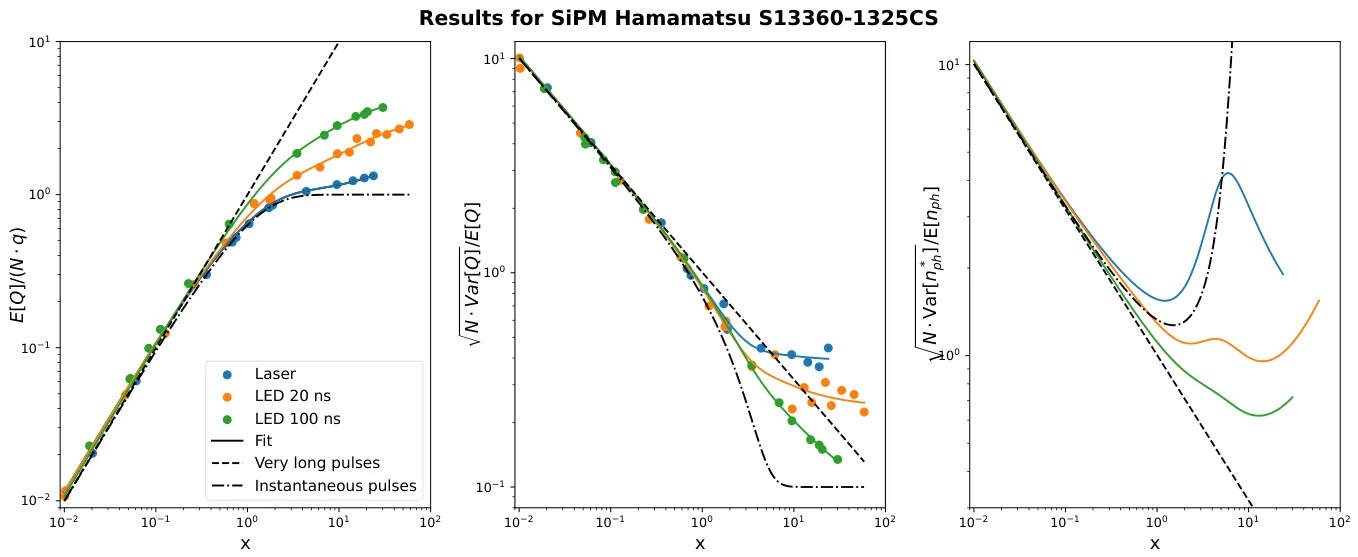
Experimental results for $\frac{E[Q]}{N\cdot q}$ and $\frac{\sqrt{N\cdot \mathrm{Var}[Q]}}{E[Q]}$ measured with the Hamamatsu S13360-1325CS SiPM using short laser pulses and rectangular LED pulses with widths of 20 and 100 ns. Fits of (27) to the laser data and of (28) to the LED data are shown in the **left-hand panel**. Fits of (30), with f(x) modeled by (33), are shown in the **middle panel**. The corresponding scaled photon-counting resolution $\frac{\sqrt{N\cdot \mathrm{Var}[n_{\mathrm{ph}}^{*}]}}{E[n_{\mathrm{ph}}]}$, computed from (39), is shown in the **right-hand panel**. The theoretical predictions for the limiting cases of instantaneous and very long light pulses are also included for comparison.

**Figure 12:**
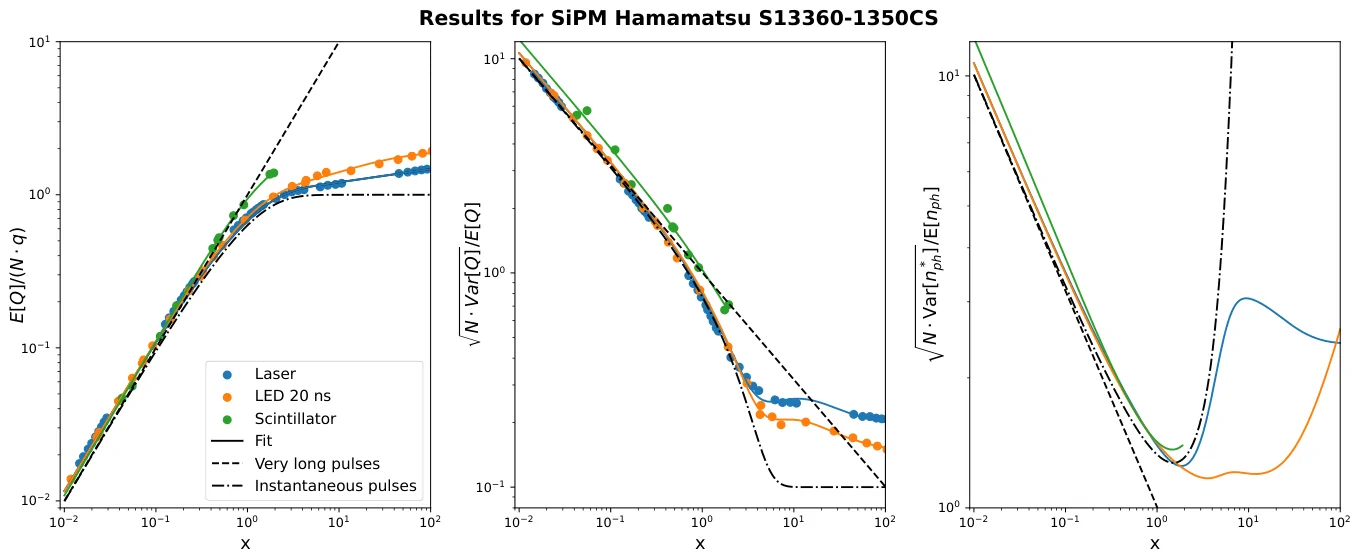
Experimental results for $\frac{E[Q]}{N\cdot q}$ and $\frac{\sqrt{N\cdot \mathrm{Var}[Q]}}{E[Q]}$ measured with the Hamamatsu S13360-1350CS SiPM using short laser pulses, rectangular LED pulses with a width of 20 ns, and a LYSO scintillator. Fits of Equation (27) to the laser and scintillation data and of Equation (28) to the LED data are shown in the **left-hand panel**. Fits of Equation (30), with f(x) modeled by Equation (33) for the laser and LED data and by Equation (A14) for the scintillation data, are shown in the **middle panel**. The corresponding scaled photon-counting resolution $\frac{\sqrt{N\cdot \mathrm{Var}[n_{\mathrm{ph}}^{*}]}}{E[n_{\mathrm{ph}}]}$, computed from Equation (39), is shown in the **right-hand panel**. The theoretical predictions for the limiting cases of instantaneous and very long light pulses are also included for comparison.

Experimental results for $\frac{E[Q]}{N\cdot q}$ and $\frac{\sqrt{N\cdot \mathrm{Var}[Q]}}{E[Q]}$ for the S13360-1325CS and S13360-1350CS SiPMs are shown in Figure 11 and Figure 12. These two SiPM models differ in several characteristics, including the number of pixels *N*, the photon detection efficiency PDE, and the gain. In addition, measurements were performed at different overvoltages ranging from 4 V to 6 V. Nevertheless, the normalized results obtained under all these conditions can be compared directly because they depend primarily on the light-pulse duration relative to the SiPM recovery time, which was 17 ns for the S13360-1325CS and 29 ns for the S13360-1350CS [24]. The incident light intensity was varied over a wide range, covering values of *x* from $10^{-2}$ to $10^{2}$, so that both the linear and nonlinear regimes were well sampled. The theoretical predictions for $\frac{E[Q]}{N\cdot q}$ and $\frac{\sqrt{N\cdot \mathrm{Var}[Q]}}{E[Q]}$ in the limiting cases of instantaneous pulses and very long pulses, in the absence of correlated noise, are also shown in the figures for comparison. For these curves, we assumed $\frac{s}{q}=0.1$ for both SiPMs.

Laser pulses were measured with both SiPMs. Although the laser pulses are much shorter than the SiPM recovery time, a non-negligible contribution from partially recovered pixels was observed, attributed to the small exponential tail in the laser pulse (see Figure 10). This effect also explains why $\frac{\sqrt{N\cdot \mathrm{Var}[Q]}}{E[Q]}$ exceeds the prediction for instantaneous light pulses at large values of *x*.

For the S13360-1325CS SiPM, rectangular LED pulses of 20 ns and 100 ns were used to investigate the effect of the light-pulse duration. As expected, the nonlinearity is weaker for the 100 ns pulses, and $\frac{\sqrt{N\cdot \mathrm{Var}[Q]}}{E[Q]}$ approaches the theoretical prediction for very long light pulses. For the S13360-1350CS SiPM, rectangular LED pulses of 20 ns were also used, while scintillation pulses from a LYSO crystal, with a decay time of 42 ns, were used to investigate an experimental case with weak nonlinearity. In this case, the maximum value of *x* was 1.9, corresponding to the highest-energy photopeak ($E_{\gamma }=1408$ keV) measured. As discussed above, the scintillation pulses exhibited super-Poissonian statistics because the light-collection efficiency depended on the γ-ray interaction position within the crystal. Consequently, $\frac{\sqrt{N\cdot \mathrm{Var}[Q]}}{E[Q]}$ lies above the theoretical predictions for Poissonian light pulses in the linear regime.

Fits of Equations (27) and (28) to the $\frac{E[Q]}{N\cdot q}$ data are shown in the figures. Equation (27) was used for the laser and scintillation data, whereas Equation (28) was used for the rectangular LED data. Nevertheless, both equations provide equally good fits for all light-pulse types. Experimental data for $\frac{\sqrt{N\cdot \mathrm{Var}[Q]}}{E[Q]}$ were fitted using Equation (30), with f(x) modeled by (33), except for the LYSO scintillator, for which f(x) was modeled by Equation (A14), including the parameter ζ. In these fits, the parameters *c*, $\frac{s}{q}$, and γ were fixed to the values measured under dark conditions, while the remaining parameters were left free. However, we imposed the constraint d=δ to reduce the number of free parameters, since these SiPMs exhibit low correlated noise. In addition, parameters that were only weakly constrained by the data were fixed to physically reasonable values. In particular, the model (33) is overparameterized to describe data obtained with the LYSO scintillator, due to the limited range of *x* that could be covered in these measurements. Consequently, the parameters β and ε were fixed to unity in this case. The results are summarized in Table 4.

The scaled photon-counting resolution $\frac{\sqrt{N\cdot \mathrm{Var}[n_{\mathrm{ph}}^{*}]}}{E[n_{\mathrm{ph}}]}$ computed from Equation (39) using the fitted models is shown in the right-hand panels of Figure 11 and Figure 12, together with the theoretical predictions for the limiting cases of instantaneous (41) and very long (40) light pulses. Notably, for laser pulses, $\frac{\sqrt{N\cdot \mathrm{Var}[n_{\mathrm{ph}}^{*}]}}{E[n_{\mathrm{ph}}]}$ reaches a minimum at x≈1.1 for the S13360-1325CS SiPM and at x≈1.3 for the S13360-1350CS SiPM; it then increases with *x* before decreasing again for x≳9 because of the small exponential tail in the laser pulse discussed above. For the LED pulses, the scaled photon-counting resolution either exhibits a local minimum or decreases monotonically, depending on the pulse width. For scintillation pulses, the highest-energy photopeak lies close to the local minimum of $\frac{\sqrt{N\cdot \mathrm{Var}[n_{\mathrm{ph}}^{*}]}}{E[n_{\mathrm{ph}}]}$. In all cases, the scaled photon-counting resolution remains above the theoretical prediction for very long light pulses, which corresponds to an ideal linear response, as expected.

## 6. Conclusions

In this work, we have investigated the statistical fluctuations in the response of passive SiPMs over their full dynamic range. Analytical expressions for the relative standard deviation of the output charge were derived for the two limiting cases of instantaneous light pulses and pulses much longer than the SiPM recovery time. These analytical formulations were then generalized to account for pixel recovery and correlated noise for arbitrary pulse durations through a phenomenological model governed by a small set of fitting parameters. Finally, the model was used to analyze the resolution in the number of impinging photons reconstructed from the measured charge.

The predictions of the proposed model for the photon-counting resolution were compared with those for the models presented in [21,31], showing that our model addresses several issues which were not properly treated previously. In particular, our model correctly accounts for the interplay of the several stochastic processes influencing the SiPM response.

The model was validated against extensive Monte Carlo simulations and experimental measurements using laser, LED, and scintillation pulses across two different SiPM devices. Excellent agreement was achieved across all operating conditions. When applied over a narrow intensity range, care must be taken to avoid model overparameterization by appropriately constraining non-essential parameters.

Our findings reveal that the photon-counting resolution is governed primarily by the number of available pixels and the light-pulse duration relative to the pixel recovery time. Correlated noise degrades performance mainly in the low-intensity regime, where secondary avalanches propagate freely; its impact progressively diminishes as saturation develops due to the lower availability of fully recovered pixels. Specifically, the resolution behavior depends on the light-pulse duration as follows:

- For pulses much shorter than the recovery time, the photon-counting resolution reaches a minimum at approximately x≈1.5 avalanche seeds per pixel before degrading rapidly due to pixel saturation.
- For pulse durations comparable to the recovery time, a broad minimum or plateau is observed over several avalanche seeds per pixel before performance deteriorates.
- For pulses much longer than the recovery time, the response remains close to linear and the photon-counting resolution improves monotonically over a significantly wider range of light intensities.

Overall, this study demonstrates that the onset of nonlinearity does not necessarily imply a degradation of photon-counting performance. On the contrary, the sub-Poissonian statistics introduced by pixel saturation partially compensate for the nonlinear compression of the SiPM response, while the suppression of correlated noise at high occupancy further improves the statistical behavior. In practical situations, the photon-counting resolution typically begins to deteriorate once the fraction of triggered pixels exceeds approximately 80%. These findings indicate that the effective dynamic range of SiPMs can therefore be significantly extended beyond the linear regime when appropriate nonlinear-response corrections are applied.

The proposed framework therefore provides a practical and computationally simple tool for predicting and optimizing the photon-counting performance of passive SiPMs over a wide dynamic range. Requiring only a minimal set of physically grounded parameters, it can be readily adapted to diverse light-pulse shapes and SiPM architectures, facilitating the optimization of high-dynamic-range SiPM instrumentation.

## Figures and Tables

**Table 1:** Fitting parameters of expression (33) (or expression (A14) for the super-Poissonian case) obtained from the simulation data without correlated noise shown in Figure 3 and Figure 4 for exponential and rectangular light pulses, respectively.

Exponential Pulses						
$T_{d}/\tau$	0.01	0.1	1	10	100	1 (sP)
*a*	0.031	0.128	1.081	9.494	91.701	1.082
*b*	0.145	0.171	0.283	0.078	0.010	0.283
α	0	0.027	0.360	0.846	0.984	0.337
β	-	0.164	0.762	1.099	1.025	0.818
ε	-	1.142	1.109	0.996	1.000	1.016
ζ	-	-	-	-	-	0.250
**Rectangular Pulses**						
$T_{r}/\tau$	0.01	0.1	1	10	100	1 (sP)
*a*	0	0.004	0.111	0.582	0.887	0.110
*b*	0	0.037	0.116	0.065	0.010	0.115
α	0	0	0.133	0.675	0.941	0.115
β	-	-	0.649	1.160	1.035	0.631
ε	-	-	0.970	0.990	0.999	0.943
ζ	-	-	-	-	-	0.250

**Table 2:** Fitted values of the parameters *c*, *d*, γ, and δ associated with correlated noise for the simulation results shown in Figure 5.

	20% Crosstalk			20% Afterpulsing		
$T_{d}/\tau$	0.1	1	10	0.1	1	10
*c*	0.237	0.237	0.237	0.079	0.079	0.079
*d*	1.324	1.198	0.007	0	0	0
γ	0.193	0.193	0.193	0.041	0.041	0.041
δ	4.193	2.812	1.398	0	0	0

**Table 3:** X-ray and γ-ray sources used in the measurements together with their corresponding photopeak energies.

Isotope	Energies (keV)
Ba-133	31, 81, 303, 356
Cs-137	662
Eu-152	40, 122, 344, 1408
Na-22	511, 1274

**Table 4:** Best-fit parameters obtained from the experimental data shown in Figure 11 and Figure 12 for the Hamamatsu S13360-1325CS and S13360-1350CS SiPMs. The normalized mean charge $\frac{E[Q]}{N\cdot q}$ was fitted using Equation (27) for the laser and scintillation data and Equation (28) for the rectangular LED data. The parameters *a* and *b* therefore have different meanings depending on the model. The scaled charge resolution $\frac{\sqrt{N\cdot \mathrm{Var}[Q]}}{E[Q]}$ was fitted using Equation (30), with f(x) given by Equation (33) for all measurements except the LYSO scintillator, for which Equation (A14) was used. The parameters *c*, $\frac{s}{q}$, and γ were fixed to the values determined from the dark-count measurements. Parameters marked with * were fixed during the fit.

	S13360-1325CS			S13360-1350CS		
Light Pulse	Laser	20 ns LED	100 ns LED	Laser	20 ns LED	LYSO
*a*	0.775	0.128	0.368	0.126	0.069	0.326
*b*	0.023	0.052	0.101	0.412	0.070	2.470
*c*	0.053	0.053	0.020	0.183	0.183	0.098
*d*	2.000 *	1.916	0.581	2.000 *	1.126	0.166
$\frac{s}{q}$	0.107	0.107	0.147	0.064	0.064	0.077
α	0.118	0.209	0.321	0.121	0.083	0.351
β	0.703	0.655	0.905	0.206	0.238	1.000 *
γ	0.044	0.044	0.034	0.131	0.131	0.077
δ	2.000 *	1.916	0.581	2.000 *	1.126	0.166
ε	2.192	1.224	0.971	1.256	1.195	1.000 *
ζ	-	-	-	-	-	0.430

## Data Availability

The data and MC code are available in the following public repository: https://github.com/VictorMoyaZ/Modeling-the-variance-of-SiPMs-in-the-nonlinear-regime-Data/tree/main (accessed on 27 August 2026).

## Abbreviations

The following abbreviations are used in this manuscript:

SiPM	Silicon photomultiplier
MC	Monte Carlo
laser	Light amplification by stimulated emission of radiation
LYSO	Lutetium–Yttrium Oxyorthosilicate
LED	Light-Emitting Diode
LiDAR	Light detection and ranging
PDE	Photodetection efficiency
NIST	National Institute of Standards and Technology

## Appendix A. Super-Poissonian Light Pulses

In many practical situations, the statistics of the incident photons deviate from a Poisson distribution and are more accurately described by a super-Poissonian model. Such statistics can be conveniently modeled by a negative binomial distribution

(A1) $$P\left(n_{\mathrm{ph}}\right)=\frac{\Gamma (n_{\mathrm{ph}}+r)}{\Gamma \left(r\right)\cdot n_{\mathrm{ph}}!}\cdot \theta^{r}\cdot {\left(1-\theta \right)}^{n_{\mathrm{ph}}},$$

with

(A2) $$\begin{array}{cc} E[n_{\mathrm{ph}}] & =\frac{r\cdot (1-\theta )}{\theta }, \end{array}$$

(A3) $$\begin{array}{cc} \mathrm{Var}[n_{\mathrm{ph}}] & =\frac{r\cdot (1-\theta )}{\theta^{2}}=\frac{E[n_{\mathrm{ph}}]}{\theta }. \end{array}$$

In this case, the unconditional distribution of the number of avalanche seeds (9) is also a negative binomial distribution with

(A4) $$\begin{array}{cc} E[n_{s}] & =PDE\cdot E[n_{\mathrm{ph}}], \end{array}$$

(A5) $$\begin{array}{cc} \mathrm{Var}[n_{s}] & =PDE\cdot E\left[n_{\mathrm{ph}}\right]\cdot \left(1+\zeta \right), \end{array}$$

where $\zeta =\frac{1-\theta }{\theta }\cdot PDE$.

For instantaneous light pulses, the expected value and variance of the number of fired pixels are given by

(A6) $$\begin{array}{cc} E[n_{f}] & =N\cdot \left(1-e^{-k_{1}\cdot x}\right), \end{array}$$

(A7) $$\begin{array}{cc} \mathrm{Var}[n_{f}] & =N\cdot \left(e^{-k_{1}\cdot x}-e^{-k_{2}\cdot x}\right)+N^{2}\cdot \left(e^{-k_{2}\cdot x}-e^{-2k_{1}\cdot x}\right), \end{array}$$

with

(A8) $$\begin{array}{cc} k_{1} & =\frac{N}{\zeta }\cdot \ln\left(1+\frac{\zeta }{N}\right)\approx 1-\frac{\zeta }{2\cdot N}, \end{array}$$

(A9) $$\begin{array}{cc} k_{2} & =\frac{N}{\zeta }\cdot \ln\left(1+\frac{2\cdot \zeta }{N}\right)\approx 2-\frac{2\cdot \zeta }{N}, \end{array}$$

where the approximate expressions correspond to the limit N≫1. Therefore, neglecting terms of order 1/N, the normalized mean response and relative charge fluctuations in the absence of correlated noise become

(A10) $$\begin{array}{cc} \frac{E[Q]}{N\cdot q} & =1-e^{-x}, \end{array}$$

(A11) $$\begin{array}{cc} \frac{\sqrt{N\cdot \mathrm{Var}[Q]}}{E[Q]} & =\sqrt{\frac{1+\frac{s^{2}}{q^{2}}+\frac{x\cdot e^{-2\cdot x}}{1-e^{-x}}\cdot \zeta }{1-e^{-x}}-1}, \end{array}$$

As expected, in the limit θ→1, the distribution (A1) tends to a Poisson distribution and ζ=0, so that (A11) reduces to (17).

In the linear regime (x≪1), as well as in the limit of very long light pulses (T≫x·τ), the above equations reduce to

(A12) $$\begin{array}{cc} \frac{E[Q]}{N\cdot q} & =x, \end{array}$$

(A13) $$\begin{array}{cc} \frac{\sqrt{N\cdot \mathrm{Var}[Q]}}{E[Q]} & =\sqrt{\frac{1+\frac{s^{2}}{q^{2}}+\zeta }{x}}. \end{array}$$

Thus, the parameter ζ, which accounts for the super-Poissonian fluctuations in the number of avalanche seeds in (A5), simply adds to the excess noise factor appearing in Equation (19).

In the saturation limit x→∞ for instantaneous light pulses (i.e., $n_{f}=N$), the SiPM response is characterized by Equations (20) and (21), which are independent of the photon statistics.

According to these results, the fitting model (33) can be adapted as follows:

(A14) $$\begin{array}{cc} f(x) & =\alpha \cdot \left(1-e^{-\beta \cdot x}\right)\cdot \left(\varepsilon +\frac{1+\frac{s^{2}}{q^{2}}+\zeta +\gamma \cdot e^{-\delta \cdot x}}{\beta \cdot x}\right)\\ \; & \;+\left(1-\alpha \right)\cdot \left(1+\frac{s^{2}}{q^{2}}+\frac{x\cdot e^{-2\cdot x}}{1-e^{-x}}\cdot \zeta +\gamma \cdot e^{-\delta \cdot x}\right)\;. \end{array}$$

In the linear regime (x≪1), this expression reduces to

(A15) $$f\left(x\right)=1+\frac{s^{2}}{q^{2}}+\zeta +\gamma .$$

In the saturation limit (x→∞), assuming δ>0, the model (A14) yields

(A16) $$f\left(x\right)=\alpha \cdot \varepsilon +\left(1-\alpha \right)\cdot \left(1+\frac{s^{2}}{q^{2}}\right),$$

which is identical to expression (36), because the signal variance is independent of the photon statistics in this limit.

This procedure can be followed to model f(x) for other photon-number distributions, provided that $E[n_{\mathrm{ph}}]$ and $\mathrm{Var}[n_{\mathrm{ph}}]$ can be obtained.
